# TTC36-Mediated Tumor Suppression via YBX3/SPRED1 Axis Paradoxically Reduces Sorafenib Sensitivity in Hepatocellular Carcinoma

**DOI:** 10.7150/ijbs.115727

**Published:** 2025-10-01

**Authors:** Wenhu Zhao, Xiangyu Ling, Kuan Li, Litao Liang, Wenbo Jia, Jinyi Wang, Yanzhi Feng, Chao Xu, Qingpeng Lv, Deming Zhu, Zhiwen Feng, Xiaoming Ai, Lianbao Kong, Wenzhou Ding

**Affiliations:** 1 Hepatobiliary Center, The First Affiliated Hospital of Nanjing Medical University, Nanjing, Jiangsu Province, China.; 2 Key Laboratory of Liver Transplantation, Chinese Academy of Medical Sciences; National Health Commission (NHC) Key Laboratory of Hepatobiliary cancers, Nanjing, Jiangsu Province, China.; 3 Department of Hepatobiliary Pancreatic Spleen Surgery, Affiliated Hospital of Jiangsu University, Zhenjiang, Jiangsu Province, China.; 4 Department of Hepatobiliary Surgery, Kunshan Hospital of Traditional Chinese Medicine, Suzhou, Jiangsu Province, China.

**Keywords:** TTC36, Hepatocellular carcinoma, mRNA stability, Sorafenib sensitivity, Precision oncology

## Abstract

**Background:** Hepatocellular carcinoma (HCC) exhibits limited therapeutic responses, partly due to undefined tumor suppressor networks. While TTC36 is downregulated in HCC and correlates with poor prognosis, its functional role, molecular mechanisms, and impact on targeted therapy remain unknown.

**Methods:** By analyzing HCC tissues RNA-seq, and scRNA-seq data of HCC tissues, we investigated the expression pattern of TTC36. The clinical relevance was analyzed by using Kaplan-Meier Plotter. Cell proliferation, migration, invasion and apoptosis were detected to confirm the function of TTC36. Mechanistic insights into TTC36-mediated HCC suppression were obtained via RNA-seq analysis, mass spectrometry analysis, molecular docking, RNA pulldown, dual-luciferase reporter assays. In animal models, the tumor growth analysis, along with IHC staining and TUNNEL staining, was used to investigate the function of TTC36 and the response to sorafenib.

**Results:** Bioinformatics and in vitro/vivo assays demonstrated TTC36 downregulation promotes HCC proliferation and correlates with poor survival. Mechanistically, TTC36 directly binds YBX3 and masks ubiquitination sites (K311/K350), inhibiting proteasomal degradation. Stabilized YBX3 enhances SPRED1 mRNA stability by binding the CACAUC motif in its 3'UTR, suppressing Ras/MAPK signaling. The TTC36/YBX3/SPRED1 axis inhibits tumor growth but induces sorafenib resistance via compensatory PI3K/Akt activation. Akt inhibition (MK-2206) reverses sorafenib resistance in TTC36-high HCC.

**Conclusion:** TTC36 is a tumor suppressor that stabilizes YBX3 to upregulate SPRED1 and inhibit Ras/MAPK-driven proliferation. Paradoxically, TTC36-high HCC develops sorafenib resistance through PI3K/Akt hyperactivation, which is overcome by combined Akt inhibition. Thus, TTC36 may serves as a predictive biomarker to stratify HCC patients for personalized therapy: sorafenib monotherapy for TTC36-low tumors and sorafenib-Akt inhibitor combination for TTC36-high, sorafenib-resistant tumors.

## Introduction

Hepatocellular carcinoma remains a leading cause of cancer-related mortality worldwide, characterized by aggressive progression and limited therapeutic options for advanced stages 1. While extensive efforts have focused on identifying oncogenic drivers of HCC, emerging evidence highlights tumor suppressor genes (TSGs) as critical regulators of tumorigenesis. TTC36 (tetratricopeptide repeat domain 36), a member of the TPR protein family, has emerged as a potential TSG with downregulation observed in multiple malignancies 2-5 often linked to promoter methylation 6. Bioinformatics analyses associate low TTC36 expression with poor prognosis in HCC 5,7. In addition, functional studies have provided initial clues: TTC36 was shown to suppress Wnt/β-catenin signaling in gastric cancer 3, and preliminary evidence in HCC suggests a potential role in apoptosis induction 8. Moreover, the paradoxical role of TSGs in modulating drug sensitivity is increasingly recognized; for instance, p53 loss can either enhance or impair drug resistance depending on cellular context 9,10. However, TTC36's role in HCC, its function in regulating cell proliferation, whether it is associated with key signaling pathways and whether TTC36 has an impact on the efficacy of target therapies remain unknown.

The Ras/MAPK pathway is a central driver of HCC proliferation, frequently hyperactivated through genetic alterations or epigenetic silencing of negative regulators 11. SPRED1 (Sprouty-related EVH1 domain-containing protein 1), a key endogenous suppressor of Ras/MAPK signaling, is downregulated in HCC 12. While mechanisms like promoter methylation or miRNA-mediated degradation contribute to SPRED1 loss 13,14, post-transcriptional control, particularly the regulation of SPRED1 mRNA stability, represents a significant unexplored area in HCC. YBX3 (Y-box binding protein 3) is an RBP (RNA-binding protein) known to stabilize target mRNAs via interaction with their 3'UTRs 15,16 and can also function as a transcription factor 17. However, the functional role of YBX3 in HCC, its regulatory mechanisms, and its potential link to critical pathways like Ras/MAPK through effectors such as SPRED1 remain largely undefined.

Here, we elucidate a novel TTC36-centered tumor-suppressive axis in HCC to directly addressing these critical knowledge gaps. TTC36 functions as a potent suppressor of HCC proliferation. Mechanistically, TTC36 directly interacts with and stabilizes the RNA-binding protein YBX3 by physically masking its key ubiquitination sites (K311 and K350), preventing proteasomal degradation. Stabilized YBX3 then, specifically binds to the CACAUC motif in the 3'UTR of SPRED1 mRNA through its cold shock domain (CSD), enhancing SPRED1 mRNA stability and protein expression. Upregulated SPRED1 effectively suppresses Ras/MAPK signaling, establishing the core TTC36/YBX3/SPRED1/Ras/MAPK axis that constrains HCC proliferation. Paradoxically, while TTC36-mediated Ras/MAPK suppression inhibits tumor growth, it concurrently induces sorafenib resistance. Mechanistically, TTC36-high HCC cells exhibit profound compensatory PI3K/Akt hyperactivation, enabling HCC cells to bypass Ras/MAPK dependency and rendering them less sensitive to sorafenib. Critically, this resistance can be effectively reversed by co-targeting Akt activation, highlighting a novel therapeutic strategy for TTC36-high, sorafenib-resistant HCC.

Collectively, this work establishes the TTC36/YBX3/SPRED1 axis as a fundamental regulator of HCC proliferation and provides critical insights into the interplay between tumor suppression and therapeutic efficacy, offering a foundation for TTC36-based patient stratification and personalized therapy (e.g., Sorafenib and Akt inhibitor combinations).

## Materials and Methods

### Clinical Samples and Cell Lines

The tissue samples in this study were obtained from HCC patients who underwent radical hepatectomy in the hepatobiliary center at the First Affiliated Hospital of Nanjing Medical University. This study was ratified by the Ethics Committee of the First Affiliated Hospital of Nanjing Medical University. All patients gave their written informed consent. The clinical features of HCC patients in this study are shown in supplementary Table S1.

Cell lines used in this study including Huh-7, MHCC97H, Hep3B, HCC-LM3, and SK-HEP-1, the human embryonic kidney cell line 293T (HEK-293T), along with the normal human liver cell line HHL-5, were acquired from the Shanghai Institute of Cell Biology, Chinese Academy of Sciences in Shanghai, China. The sorafenib-resistant Hep3B cell line was previously established by our research group, while the sorafenib-resistant SK-HEP-1 cell line was purchased from WanWu Biotechnology (Anhui, China).

### Cell Culture

All cell lines mentioned above were cultured with DMEM (Dulbecco's modified Eagle's medium) (Gibco, CA, USA). The media were supplemented with 10% bovine serum (WISENT, Canada) and 1% penicillin/streptomycin (Gibco, CA, USA). All cell lines were grown in a humidified cell incubator (37 °C, 5% CO_2_).

### Quantitative Real-Time PCR (qRT‒PCR)

The RNA extraction kit (Invitrogen) was employed to carry out the total RNA extraction. The NanoDrop 2000 spectrophotometer (NanoDrop Technologies, MA, USA) was utilized to determine the concentration of RNA. The extracted RNA was reverse transcribed into cDNA with the Prime Script RT kit (TaKaRa, Dalian, China). Quantitative real-time polymerase chain reaction (qRT-PCR) was conducted using the SYBR Premix ExTaq II (TaKaRa) and the ABI 7900 PCR system (Applied Biosystems, CA, USA). The sequence of specific primer used in this study is listed in supplementary Table S2.

### Western Blot

Radioimmunoprecipitation assay (RIPA) buffer solution containing 1 mM phenylmethylsulfonyl fluoride (PMSF) was used to extract total proteins. The proteins were separated using sodium dodecyl sulfate-polyacrylamide gel electrophoresis (SDS-PAGE) and transferred to polyvinylidene fluoride (PVDF) membranes (Bio-Rad, CA, USA). The membranes were blocked with 5% bovine serum albumin (BSA) for 2 hours, followed by incubating with primary antibodies at 4 °C overnight. Next, using TBST buffer, the membranes were washed 3 times and incubated with HRP-conjugated secondary antibodies for 2 h at normal room temperature. Protein quantification was carried out using hypersensitive ECL exposure solutions and Image Lab software (Bio-Rad, Hercules, CA, USA). The specific antibodies employed in this experiment are listed in Supplementary Table S3.

### Transfection

In order to construct the stable TTC36-overexpressed Hep3B and TTC36-knockdown-SK-HEP-1 cell lines, the human TTC36 overexpression plasmid and lentivirus-sh-TTC36 were purchased from Genomeditech (Shanghai, China). Plasmid transfections were carried out using Lipofectamine 3000 (Invitrogen). For lentiviral transfections, polybrene (Invitrogen) was utilized to enhance transfection efficiency. Puromycin (Invitrogen) was employed for selecting stable transfected cells. The shRNA sequences used in this work are listed in Supplementary Table S4.

### Cell Counting Kit-8 (CCK-8) Assay

Transfected cells were placed into each well of a 96-well plate with a density of 1000 cells per well. After that, the Cell Counting Kit-8 (APEBIO, USA) was used according to manufacturer's instructions. The absorbance of each group was determined at a wavelength of 450 nm for each 24 hours.

### Colony Formation Assay

1,000 individual HCC cells were seeded in 6-well plates and incubated in a cell culture incubator for 14 days. Colonies were fixed with paraformaldehyde and stained with crystal violet stain (Beyotime, China). After drying, the colonies were photographed and counted using Image J.

### 5-Ethynyl-20-deoxyuridine (EdU) Assay

The EdU assay was conducted using an EdU Kit from Ribobio. Cells were placed in a 24-well plate and exposed to an EdU solution for 2 hours. Following fixation with 4% paraformaldehyde, cell permeabilization was achieved using Triton X-100. Cells were then stained with the Apollo solution, and the nuclei were marked with DAPI. Images were taken using a fluorescence microscope (DM4000B-1, Leica, Frankfurt, Germany).

### Cell Cycle Assay

The cell cycle assay was carried out following the protocol provided in the cell cycle staining kit (MultiSciences Biotech, China, CCS012). In summary, one million cancer cells were harvested, washed twice with phosphate-buffered saline (PBS), and centrifuged at 1000 rpm for 5 minutes. The cells were then resuspended in 1 ml of DNA staining solution along with 10 µl of Permeabilization solution and incubated for 30 minutes at room temperature, shielded from light. The samples were analyzed using a flow cytometer. Data analysis was performed using FlowJo v10.0 (FlowJo, LLC).

### Transwell Assay

In the migration assay, a total of 2x10^4^ transfected cells were introduced into the upper chambers of Transwell plates, which contained 200 µl of serum-free medium. The lower chambers were filled with 400 µl of complete medium. Following a 48-hour incubation period, the cells were stained with crystal violet. The cells that remained in the upper chamber were discarded, and microscopic imaging was performed. In the invasion assay, the same procedures as the migration assay were followed, with the exception that 50 µl of Matrigel (BD Biosciences, NJ, USA) was added to the upper chamber.

### Wound Healing Assay

Transfected cells were cultured in a 6-well plate until they formed a monolayer covering the entire bottom of each well. To create a wound on the cell monolayer, a 200-µL pipette tip was used. The process of wound healing was observed and recorded at both 0 and 24 hours.

### RNA sequencing

Hep3B cells transfected with TTC36-overexpression plasmid or vector were harvested and exposed to TRIzol. The lysates were sent to Tsingke Biological Technology (Beijing, China) with solid carbon dioxide, then RNA sequencing was carried out.

### Subcutaneous Tumor Model

Four-week-old male BALB/c nude mice were sourced from Vital River (Beijing, China) and randomly divided into four groups, each containing five mice. The transfected cells were suspended in PBS and injected subcutaneously into the flanks of the mice. Tumor size was monitored every 4 days. After four weeks, the mice were sacrificed, and the volume and mass of the subcutaneous tumors were measured. Ethical approval for all animal experiments was obtained from the Institutional Animal Care and Use Committee (IACUC) at the First Affiliated Hospital of Nanjing Medical University. All procedures involving animals adhered to the operating guidelines set forth by the IACUC.

### Immunoprecipitation and Silver Staining

PBS that had been precooled was employed to wash HCC cells, and afterwards, NP-40 lysis solution (Beyotime, Shanghai, China) was utilized to extract the total proteins of HCC cells. After pre-clearing with Protein A/G PLUS-Agarose (SC-2003, Santa Cruz Biotechnology, CA, USA), cell lysates were immunoprecipitated with the indicated antibodies. The purified immunoglobulin G (IgG) (12370, Sigma-Aldrich, St. Louis, MO, USA) derived from the host species was used as a negative control. Immunocomplexes were obtained using Protein A/G PLUS-Agarose. The immunoprecipitated proteins were combined with SDS-PAGE loading buffer and then eluted by boiling, and later detected by western blotting. The silver staining of SDS-PAGE gel was performed using the Fast Silver Stain Kit (Beyotime) following established protocols.

### Mass Spectrometry Analysis

Proteins obtained by immunoprecipitating with TTC36 antibody and IgG were sent to Cosmos Wisdom Biotech Co., Ltd. (Hangzhou, China), then the mass spectrometry analysis was carried out.

### Coimmunoprecipitation (Co-IP) Assay

Cells were harvested at 80% confluence and lysed in ice-cold lysis buffer for 30 minutes on ice. Cell lysates were centrifuged at 12,000 x g for 15 minutes at 4 °C to remove debris, then incubated with 2 µg of primary antibody overnight at 4C with gentle rotation. Subsequently, 30 µL of protein A/G agarose beads were added and incubated for an additional 2 hours at 4 °C. Beads were washed five times with lysis buffer to remove non-specifically bound proteins. Bound protein were eluted by boiling the beads in 2X SDS sample buffer for 5 minutes. Finally, the Western blot was carried out observe the protein interaction.

### RNA Stability Assays

Actinomycin D (Sigma-Aldrich, U.S.) was used to block the transcription process, with the working concentration 2 μg/mL. After treatment, cell samples were collected at 3 h and 6 h, then RNA was extracted for qRT-PCR analysis.

### RNA Pulldown Assay

The full-length and antisense of SPRED1 mRNA were supplied by RiboBio. A pulldown assay was carried out with a pulldown kit (Thermo Scientific, USA). Magnetic beads were utilized to immobilize the RNAs, and these were then combined with the RNA-binding buffer and incubated at room temperature for 30 minutes. The supernatant was taken away, and the resultant precipitate was incubated with the cell lysate at 4 °C for 1 hour. Protein extraction was conducted using an elution buffer, and the bound proteins were identified through Western blot analysis.

### RIP Assay

An RNA immunoprecipitation kit (Geneseed, Guangzhou, China) was employed to carry out RIP experiments in accordance with the supplied protocols. To sum up, the cell lysate was incubated overnight along with magnetic beads and either the anti-YBX3 or the anti-Flag. Thereafter, protease K was used on the magnetic beads to remove the proteins. Eventually, qRT-PCR was utilized to assess the separated RNA.

### Dual-Luciferase Reporter Assay

The reporter gene plasmid, provided by IBSBIO (Shanghai, China), was used for detection following the kit's instructions. After transfecting HEK 293T cells, cell lysates were obtained and centrifuged at 10,000-15,000 rpm for 3-5 minutes. The supernatant was then transferred to a new EP tube for analysis. According to the instrument manual, fluorescence detection was performed by adding 20 μL of the sample to 20 μL of Firefly Luciferase Assay Reagent, mixing thoroughly, and measuring the RLU. Subsequently, 20 μL of Renilla Luciferase Assay working solution was added to the mixture, and the RLU was measured again. The reporter gene activation level was determined by the ratio of the sample RLU to the standard RLU value.

### Analysis of Apoptosis

The apoptotic rate was determined by an Apoptosis Detection Kit (BD, USA). Annexin V-FITC binds to outward phosphatidylserine in the early stage of apoptosis. In addition, necrotic or late apoptotic cells can be stained by PI. Briefly, cells were harvested and stained with Annexin V-FITC and PI at room temperature. The apoptotic rate was determined and analyzed by BD FACS Calibur (BD, USA) and FlowJo software.

### Statistics Analysis

The data obtained from the experiment carried out in this research were analyzed by means of SPSS software version 26.0 (IBM, SPSS, Chicago, IL, USA) and GraphPad Prism version 9.0 (GraphPad, San Diego, CA, USA). Statistical analysis was carried out to determine the differences between two groups via the Student's t-test. For comparisons among more than two groups, one-way analysis of variance was utilized. The clinical characteristics were evaluated with the chi-square test, and correlation analysis was conducted through the Spearman correlation test. Data is presented as mean ± standard deviation (SD). * *P* < 0.05, ** *P* < 0.01, *** *P* < 0.001.

## Results

### TTC36 is Downregulated in HCC Tissue and Correlates with Poor Prognosis

**Figure 1:**
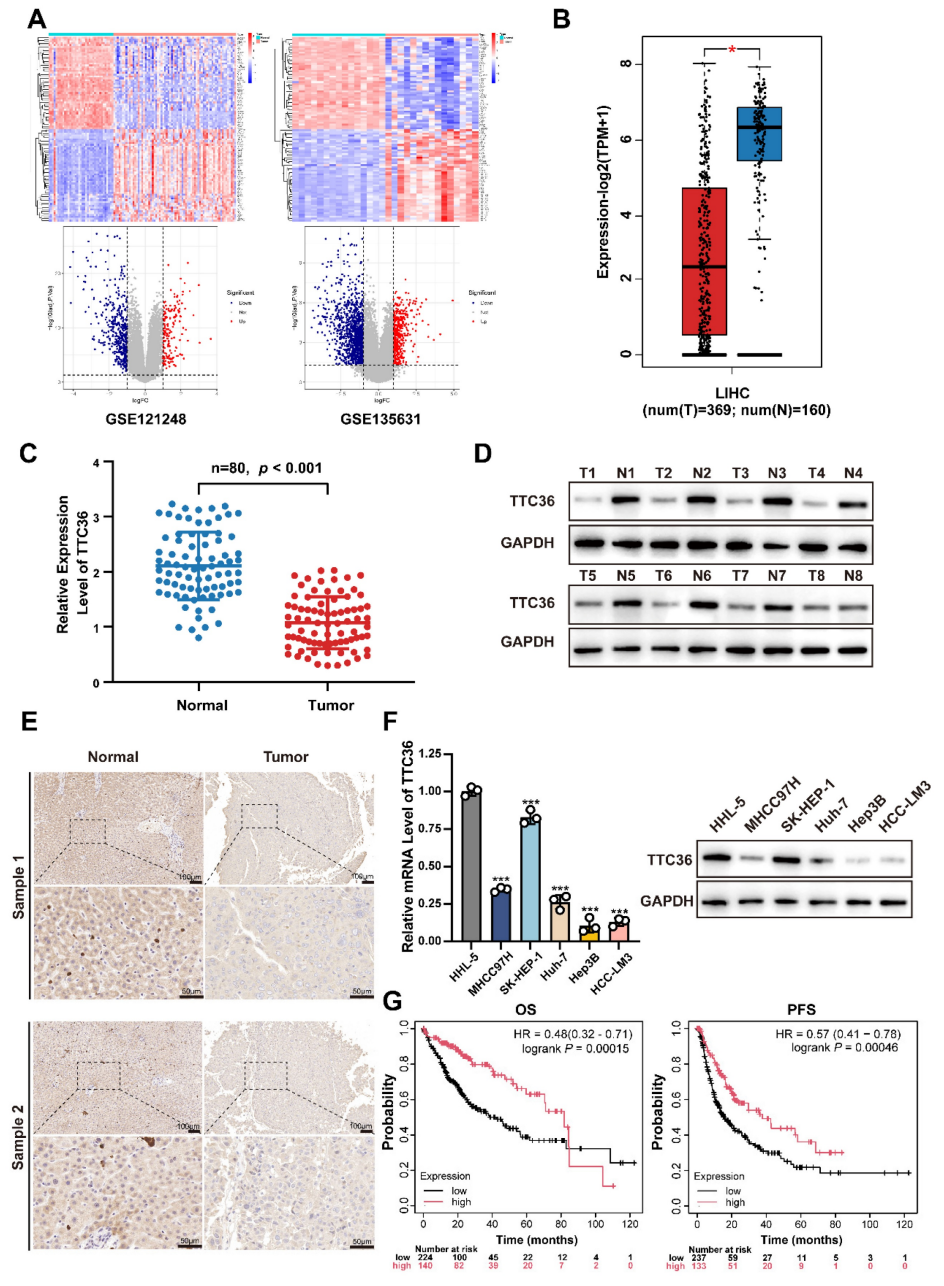
TTC36 is downregulated in HCC tissue and correlates with poor prognosis. **A**. Heatmaps and volcano plots of differentially expressed genes between tumor and adjacent non-tumor tissue of HCC (GSE121248: Normal vs. Tumor = 37 vs. 70; GSE135631: Normal vs. Tumor = 15 vs. 15; Criteria: adjusted *P* value < 0.05, |log2FC| > 1). **B**. TTC36 expression level comparison in LIHC based on TCGA data (T=369, N=160). **C**. Quantitative RT-PCR analysis of TTC36 mRNA expression in 80 paired HCC tumor (T) and adjacent non-tumor (N) tissues (n=80, *P* < 0.001). **D**. Western blot analysis of TTC36 protein expression in 8 representative paired HCC samples (T: tumor tissue, N: non-tumor tissue). **E**. Immunohistochemical staining of TTC36 in paired HCC tissues. Representative images show TTC36 expression in non-tumor tissues (left) and tumor tissues (right). Scale bar: 10×magnification: 100 μm; 40×magnification: 50 μm. **F**. TTC36 expression in normal hepatocytes (HHL-5) and five HCC cell lines (SK-HEP-1, MHCC97H, Huh-7, HCC-LM3, and Hep3B) as determined by western blotting and qRT-PCR. **G**. Kaplan-Meier survival analysis of HCC patients stratified by TTC36 expression levels (high vs. low, OS, *P* = 0.00014; PFS, *P* = 0.00046).

To delineate transcriptomic alterations in hepatocellular carcinoma (HCC), we analyzed two independent GEO cohorts: GSE121248 and GSE135631 using thresholds |log2FC| > 1 and adjusted *P* value < 0.05 (Fig. 1A). Cross-dataset intersection revealed 94 conserved upregulated and 309 suppressed genes shared between both datasets Fig. S1A). After systematically screening the functional annotations and existed literature reports of 309 down-regulated differentially expressed genes, TTC36 captured our attention as a ​poorly characterized putative tumor suppressor​ for HCC that ​warrants in-depth mechanistic investigation.

Next, the downregulation of TTC36 in HCC was systematically validated through multi-platform genomic interrogation. Cross-validation using GEPIA database demonstrated significantly lower TTC36 expression in HCC tissues compared to normal liver tissues (Fig. 1B) (18. Strikingly, pan-cancer analysis revealed that TTC36 downregulation was not restricted to HCC but was also observed in other malignancies, suggesting its broad involvement in carcinogenesis Fig. S1B) (19.

To address the expression pattern of TTC36 in HCC tissues, we performed single-cell RNA sequencing (scRNA-seq) analysis of paired HCC and adjacent non-tumorous tissues using publicly available datasets GSE282701. The result revealed that TTC36 is predominantly expressed in hepatocytes (both normal hepatocytes in adjacent tissues and HCC cells in tumor tissues), with negligible expression in other cell types. In addition, the result showed that TTC36 expression is markedly higher in normal hepatocytes compared to HCC cells, both in terms of total and average expression level Figure S1C-F). These findings suggest that TTC36 is primarily sourced from hepatocytes.

Next, the expression level of TTC36 was further verified via qRT-PCR in an expanded cohort of 80 paired HCC tumor and para-tumor tissues (Fig. 1C). Meanwhile, western blotting in representative cases also confirmed this expression pattern (Fig. 1D). Immunohistochemical staining of 8 paired samples further demonstrated marked depletion of TTC36 protein in malignant hepatocytes compared to adjacent normal parenchyma, with representative images shown in Fig. 1E.

In addition, the TTC36 expression was also determined in normal hepatocytes (HHL-5) and five HCC cell lines. Consistent with the clinical observations, all HCC cell lines exhibited marked TTC36 suppression compared to normal hepatocytes (Fig. 1F). Besides, the results showed highest TTC36 abundance in SK-HEP-1 and the lowest in Hep3B. Therefore, we selected SK-HEP-1 and Hep3B for subsequent experiments.

Next, to assess the clinical prognostic value of TTC36, we used Kaplan-Meier plotter integrating cancer data from TCGA, GEO, and EGA to generate OS (364 cases) and PFS (370 cases) curves. The result revealed that high TTC36 expression correlated with significantly prolonged overall survival (OS) and progression-free survival (PFS; Fig. 1G).

Additionally, we investigated the relationship between TTC36 expression and immune cell infiltration in HCC. Low TTC36 expression correlated with reduced infiltration of B cells, CD4⁺ T cells, dendritic cells, and neutrophils (Fig. S1G), suggesting TTC36 may function as a potential regulator of the tumor immune microenvironment. we analyzed the correlation between TTC36 expression and immune checkpoint molecules using the HCC data from TCGA database. The results revealed that TTC36 expression shows a significant negative correlation with immune checkpoint molecules PD-1, CTLA-4, TIM-3, and TIGIT (Figure S1H), indicating its potential involvement in immune microenvironment remodeling.

Collectively, TTC36 emerges as a ​predominantly hepatocyte-expressed putative tumor suppressor​ in HCC, where its downregulation is consistently associated with poor clinical outcomes.

### TTC36 Knockdown Promotes HCC Cell Proliferation *In vitro* and *In vivo*

**Figure 2:**
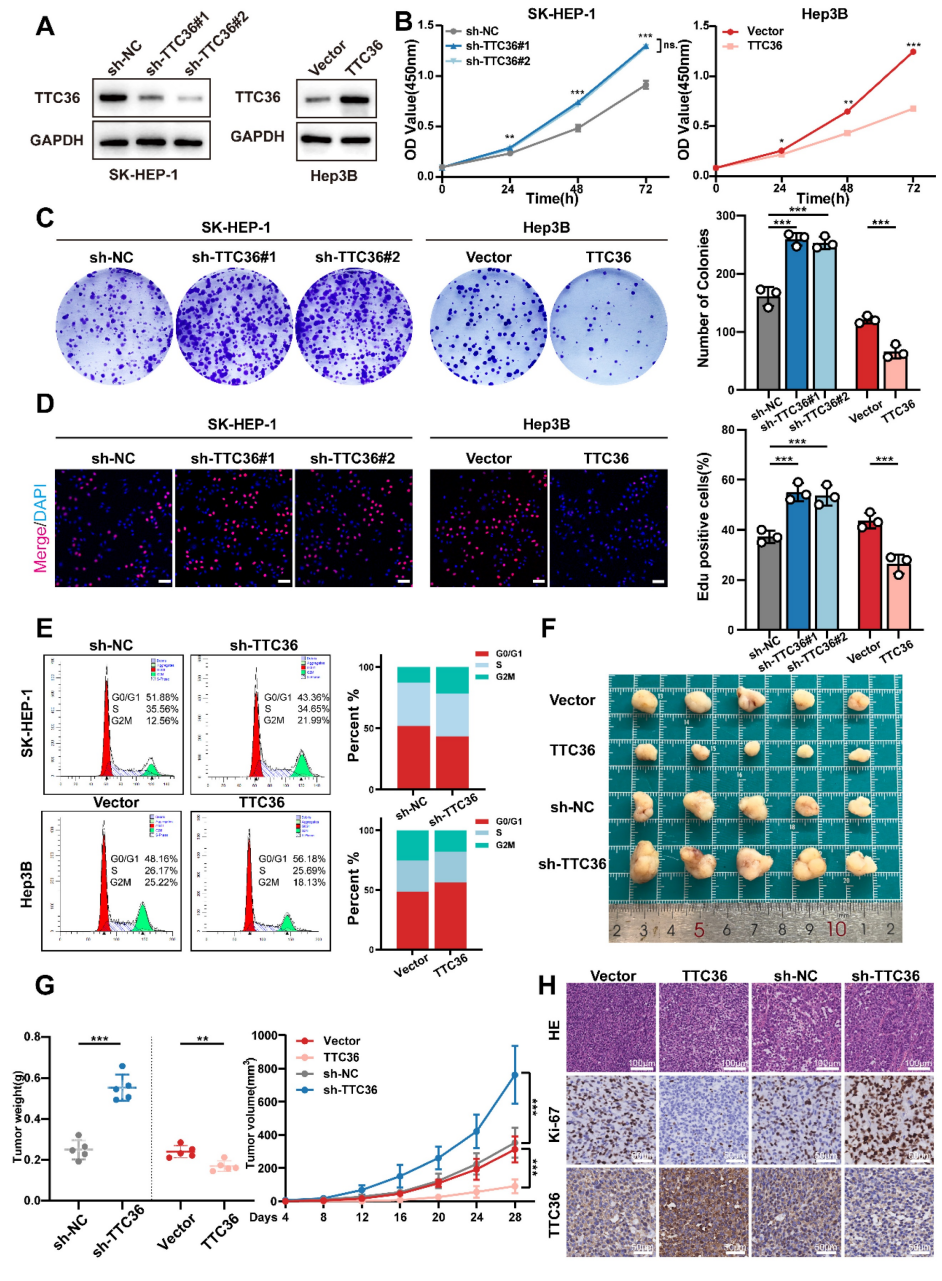
Suppression of TTC36 promotes HCC cells proliferation both *in vivo* and *in vitro.* **A**. Western blot analysis of TTC36 knockdown and overexpression efficiency in SK-HEP-1 cells and Hep3B cells. **B**. CCK-8 assays assessing the proliferative capacity of SK-HEP-1 cells with TTC36 knockdown and Hep3B cells with TTC36 overexpression. **C**. Colony formation assays showing the impact of TTC36 knockdown and overexpression on colony-forming ability. **D**. EdU incorporation assays demonstrating DNA synthesis in TTC36-modulated cells (merge images, scale bar = 50μm. Images of DAPI and EdU are shown in Figure. S2). **E**. Flow cytometry analysis of cell cycle distribution in TTC36-modulated cells. **F**. Images of subcutaneous xenografts tumors from nude mice injected with TTC36-modulated HCC cells. **G**. Tumor volumes and weights analysis. **H**. HE and immunochemical staining (Ki-67, TTC36) of the xenograft tumors (scale bar = HE: 100μm, IHC: 50μm).

To elucidate the functional significance of TTC36 in HCC progression, sh-RNAs and an overexpression plasmid were employed to construct TTC36-knockdown SK-HEP-1 cells and TTC36-overexpressed Hep3B cell lines. The efficacies of sh-RNAs and TTC36-overexpression plasmid were verified by qRT-PCR (Fig. S2A) and Western blotting (Fig. 2A). Finally, we selected the sh-TTC36#1 and the sh-TTC36#2 for subsequent functional assays based on their robust knockdown efficiency.

Functional assays demonstrated that TTC36 plays a critical role in regulating HCC cell proliferation. CCK-8 assays showed TTC36 knockdown promoted SK-HEP-1 proliferation, whereas overexpression inhibited it in Hep3B cells. (Fig. 2B). Consistent with these findings, TTC36 knockdown increased colony formation, whereas overexpression reduced it (Fig. 2C). EdU assays also showed increased DNA synthesis in knockdown cells and decreased synthesis in overexpressing cells (Figs. 2D, S2B). Flow cytometry indicated TTC36 knockdown increased the G2/M phase population, while overexpression decreased it (Fig. 2E), suggesting a key role in cell cycle progression.

While TTC36 levels significantly affected HCC cell proliferation, subsequent assays showed no detectable impact on apoptosis, migration, or invasion (Fig. S2C-E). Therefore, we focused specifically on elucidating TTC36's regulatory role in HCC proliferation.

To validate these findings *in vivo*, we established subcutaneous xenograft and pulmonary metastasis models in nude mice. In subcutaneous xenografts, TTC36 overexpression significantly reduced tumor volume/weight and Ki-67 levels, while knockdown increased both versus control group after 28 days (Fig. 2F-G). Conversely, pulmonary metastasis models showed no significant differences in TTC36-modified groups compared to their control groups (Figure S2F), consistent with *in vitro* invasion assays.

Collectively, these results demonstrate that TTC36 downregulation promotes tumor proliferation both *in vitro* and *in vivo*.

### TTC36 Represses the Ras/MAPK Signaling Pathway through SPRED1 Upregulation

**Figure 3:**
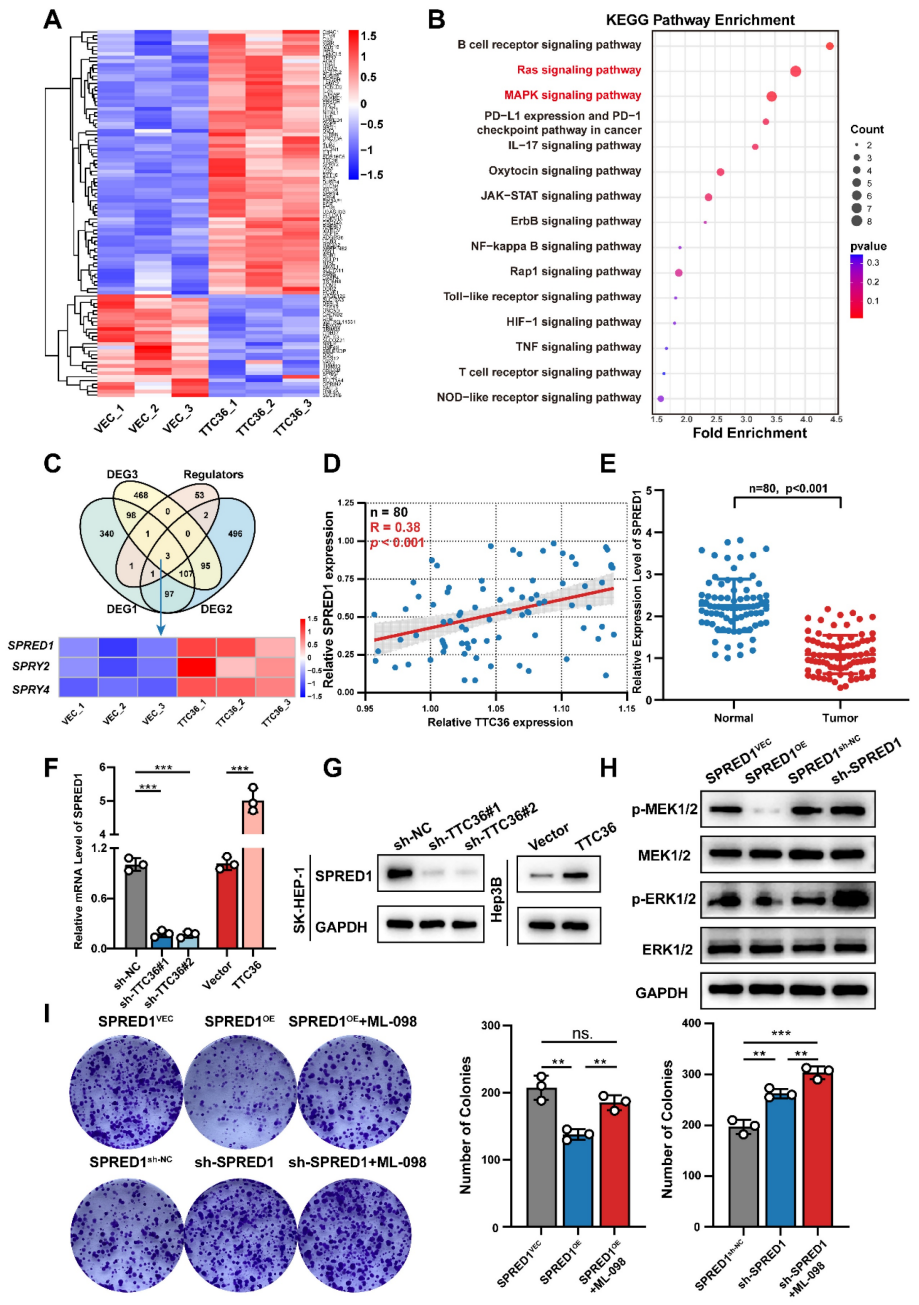
TTC36 suppresses HCC progression via SPRED1-mediated inhibition of Ras/MAPK signaling. **A**. Hierarchical clustering heatmap of RNA-seq data from TTC36-overexpressing and control HCC cells. **B**. KEGG pathway enrichment analysis of differentially expressed genes. Top 15 pathways ranked by significance. **C**. Venn diagram identifying SPRED1, SPRY2, and SPRY4 as overlapping genes between DEGs and Ras/MAPK regulators. **D**. Correlation analysis of TTC36 and SPRED1 mRNA levels in HCC clinical samples (n=80, R=0.4059, *P* = 0.0057). **E**. SPRED1 expression level in 80 paired HCC and non-tumor tissues.** F-G**. SPRED1 mRNA (**E**) and protein (**G**) levels in TTC36-modulated cells. **H**. Western blot analysis of MEK/ERK phosphorylation in SPRED1-modulated cells. **I**. Colony formation assays of SPRED1-modulated cells treated with Ras/MAPK activator ML-098.

To identify downstream targets through which TTC36 inhibits HCC cell proliferation, we performed RNA-seq on Hep3B cells overexpressing TTC36 or empty vector. Differentially expressed genes are visualized in the heatmap (Fig. 3A). Subsequent KEGG pathway enrichment analysis revealed Ras and MAPK signaling pathways as the second and third most enriched pathways, respectively (Fig. 3B). Notably, these pathways share overlapping components and are critically involved in tumor proliferation. GSEA further confirmed significant downregulation of both pathways in TTC36-overexpressing cells compared to controls (Fig. S3A). Based on these findings, we hypothesized that TTC36 regulates HCC proliferation through the Ras/MAPK pathway. To investigate the mechanism, we intersected DEGs with known Ras/MAPK regulators, identifying three Sprouty-family genes (SPRED1, SPRY2, and SPRY4) - established suppressors of Ras signaling (11 (Fig. 3C). We then examined the TTC36-SPRED1/SPRY2/SPRY4 correlation in clinical HCC specimens. Only SPRED1 expression positively correlated with TTC36 (Fig. 3D, S3B). Further analysis revealed significant downregulation of SPRED1 in HCC tumors versus paired adjacent tissues (Fig. 3E). Consistent with this, TCGA Kaplan-Meier survival analysis demonstrated poorer survival (shorter OS, PFS) in patients with low SPRED1 expression Fig. S3C). Therefore, SPRED1 was selected as the downstream target mediating TTC36's suppression of HCC proliferation. Consistent with mRNA-seq data, both qPCR and WB confirmed TTC36 overexpression upregulates SPRED1, while knockdown suppresses it (Fig 3F-G). Given SPRED1's established role in inhibiting Raf-mediated MEK/ERK activation to suppress Ras/MAPK signaling (12, we assessed downstream phosphorylation in HCC cells. The result showed SPRED1 overexpression significantly reduced MEK1/2 and ERK1/2 phosphorylation, whereas knockdown enhanced it compared to controls (Fig 3H). Concurrently examining two other Ras/MAPK branches (p38 and JNK), we found SPRED1 modulation did not alter their phosphorylation Fig. S3D). This confirms SPRED1 specifically suppresses Ras/MAPK signaling via MEK/ERK inhibition in HCC cells.​ Colony formation assays demonstrated that SPRED1 overexpression significantly inhibited Hep3B cell proliferation, and this effect was reversed by the Ras/MAPK agonist ML-098 (Fig. 3I), further supporting SPRED1's role in inhibiting HCC proliferation via Ras/MAPK suppression.

Collectively, these data establish initial evidence that TTC36 inhibits HCC proliferation by upregulating SPRED1 to suppress the Ras/MAPK pathway.

### TTC36 Interacts with YBX3 to Regulate SPRED1 Expression

**Figure 4:**
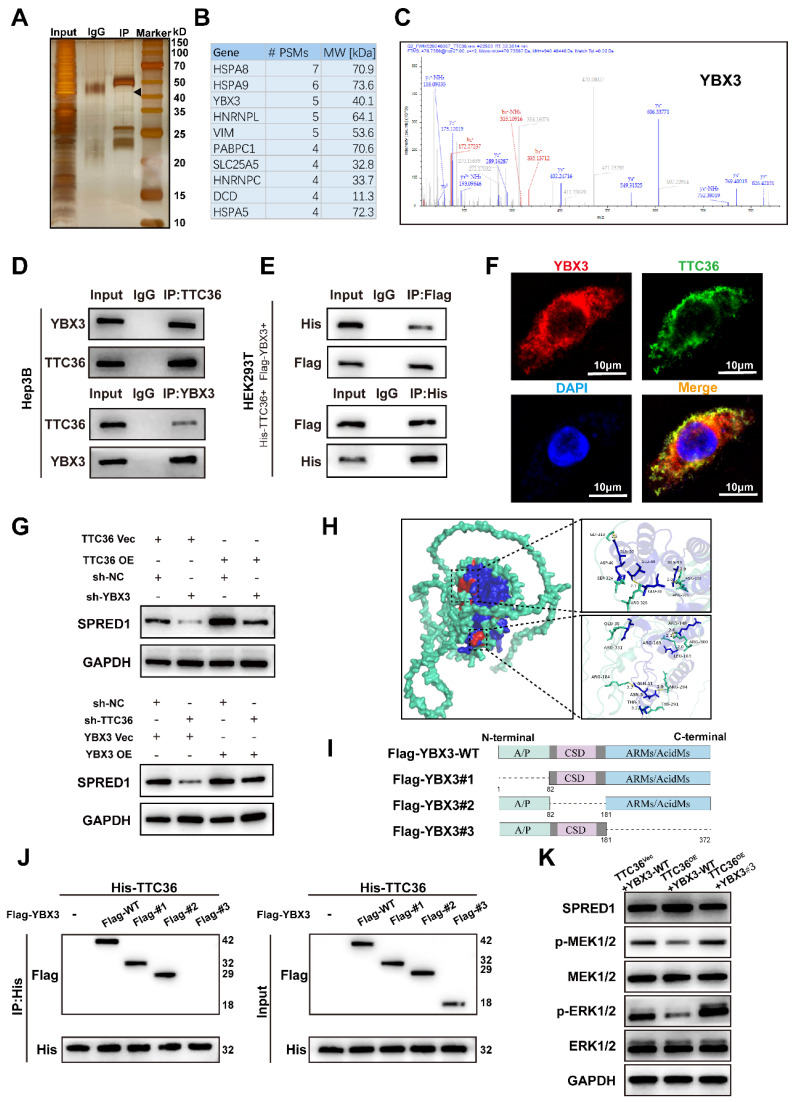
TTC36 interacts with YBX3 to regulate SPRED1 expression and Ras/MAPK signaling. **A**. Silver staining of anti-TTC36 immunoprecipitates from TTC36-overexpressing Hep3B cells. Arrow indicates YBX3 band. **B.** Top 10 candidates ranked by PSM value based on mass spectrometry analysis results. **C**. Representative MS/MS spectrum of YBX3 peptide. **D**. Endogenous Co-IP of TTC36 and YBX3 in Hep3B cells. **E**. Exogenous Co-IP in 293T cells transfected with His-TTC36 and Flag-YBX3. Blots probed with anti-His/Flag antibodies. **F**. Immunofluorescence showing cytoplasmic co-localization of TTC36 (green) and YBX3 (red). Nuclei: DAPI (blue). Scale bar: 10μm. **G**. Western blot of SPRED1 in TTC36-modulated cells with YBX3 knockdown or overexpression. **H**. Predicted TTC36-YBX3 interaction interface from molecular docking (Green: TTC36; Blue: YBX3; Red: interaction face). **I**. The diagrammatic sketch of FLAG-tagged WT or truncated mutant plasmids of YBX3. **J**. Co-IP analysis of TTC36 with YBX3 truncation mutants. **K**. SPRED1 expression and Ras/MAPK activation in cells expressing YBX3-WT or ARMs/AcidMs-deleted mutant (Flag-YBX3#3).

To investigate the mechanism by which TTC36 regulates SPRED1 expression, the eluted TTC36 protein complex obtained via immunoprecipitaion was denatured and separated by SDS-PAGE and visualized by silver staining (Fig. 4A). Mass spectrometry (MS) analysis was further performed to identify potential TTC36-interacting proteins. Among these, YBX3 (Y-box binding protein 3)— an RNA-binding protein involved in mRNA stability (15,16 and translational promotion 20 — captured our attention, as its apparent molecular weight on SDS-PAGE converged with the theoretical molecular weight of YBX3 identified by MS analysis (Fig. 4B-C).

Multiple lines of evidence confirmed the TTC36-YBX3 interaction. Endogenous co-immunoprecipitation (Co-IP) in Hep3B cells established their association under physiological conditions. Direct binding was further demonstrated by exogenous Co-IP using His-TTC36 and Flag-YBX3 expressed in HEK293T cells. Additionally, immunofluorescence microscopy revealed significant cytoplasmic co-localization of TTC36 and YBX3, providing spatial evidence for their interaction (Fig. 4D-F).

Functional rescue experiments demonstrated that TTC36 acts through YBX3 to regulate SPRED1. Overexpression of TTC36 increased SPRED1 levels, an effect reversed by co-transfection of sh-YBX3. Conversely, overexpression of YBX3 rescued the decrease in SPRED1 expression induced by sh-TTC36. These results indicate that TTC36 regulates SPRED1 expression by recruiting YBX3 (Fig. 4G).

To figure out the TTC36-YBX3 interaction domain, molecular docking predicted 15 hydrogen bonds between the proteins, with 8 located in YBX3's ARMs/AcidMs domain (Fig. 4H, S4A). Subsequent mapping assays using full-length or truncated YBX3 constructs revealed TTC36 co-immunoprecipitated with wild-type YBX3 and truncations #1 and #2, but not #3, indicating the ARMs/AcidMs domain mediates binding (Fig. 4I-J).

We then tested functional consequences for SPRED1 and Ras/MAPK signaling. Compared to control (TTC36 empty vector + wild-type YBX3), co-transfection of TTC36 and wild-type YBX3 increased SPRED1 expression and suppressed MEK/ERK activation. This effect was partially reversed when TTC36 was co-expressed with the non-binding YBX3#3 mutant, demonstrating that TTC36-YBX3 binding promotes SPRED1 expression and inhibits Ras/MAPK signaling (Fig. 4K).

Collectively, these results demonstrated that TTC36 upregulates SPRED1 through binding YBX3 via its ARMs/AcidMs domain, thereby suppressing Ras/MAPK signaling.

### TTC36 Stabilizes YBX3 by Masking Ubiquitination Sites

**Figure 5:**
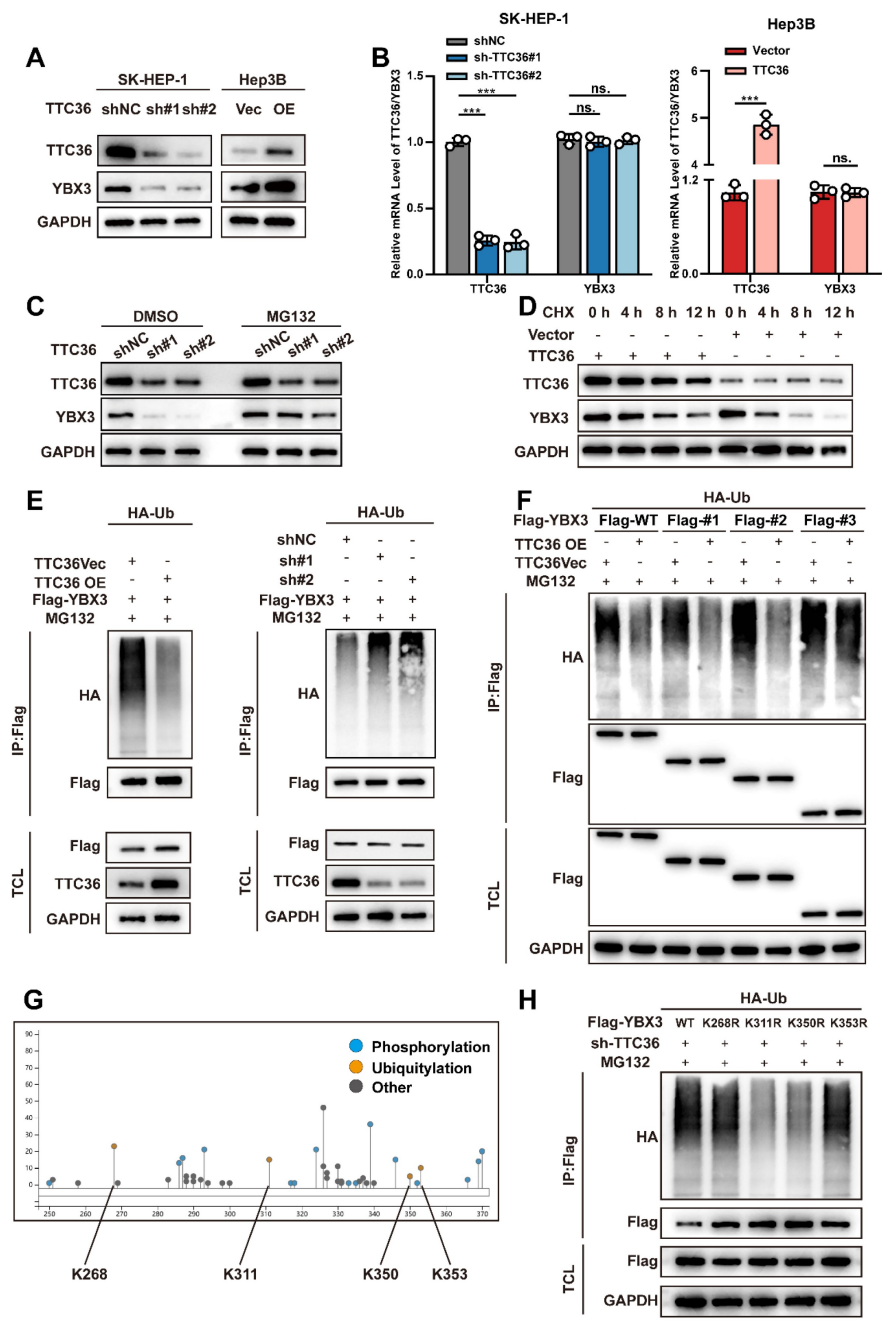
TTC36 stabilizes YBX3 by masking ubiquitination sites. **A.**‌ Western blot of YBX3 protein in TTC36-modulated SK-HEP-1 and Hep3B cells. ‌**B.**‌ qRT-PCR analysis of YBX3 mRNA levels. ‌**C.**‌ YBX3 recovery in TTC36-knockdown SK-HEP-1 cells treated with MG-132. ‌**D.**‌ Cycloheximide chase assay of YBX3 half-life extension by TTC36 overexpression in Hep3B cells. ‌**E.**‌ Ubiquitination assays of YBX3 in TTC36-modulated cells (TCL: total cell lysate) ‌**F.**‌ Ubiquitination levels of YBX3 truncation mutants with or without TTC36 overexpression. ‌**G.**‌ Predicted ubiquitination sites within the YBX3 ARMs/AcidMs domain. ‌**H.**‌ Ubiquitination profiles of YBX3 lysine mutants (K-to-R substitutions).

Previous studies indicated TTC36 stabilizes HBP by inhibiting its interaction with the PELI1 E3 ubiquitin ligase 21. To determine whether TTC36 similarly regulates YBX3, we assessed its effect on YBX3 mRNA and protein levels. Knockdown or overexpression of TTC36 altered YBX3 protein levels, but not mRNA levels (Fig. 5A-B), suggesting post-translational regulation. Ubiquitination modification is a well-known posttranslational modification of proteins through which protein will be degraded after marked by ubiquitin. Given the critical role of dysregulated ubiquitin-mediated proteasomal degradation in tumorigenesis 22, we investigated whether TTC36 regulates YBX3 by affecting its ubiquitination. To address this, we treated shTTC36-transfected SK-HEP-1 cells with the proteasome inhibitor MG132 and found that the decrease in YBX3 protein levels induced by TTC36 knockdown was rescued (Fig. 5C). Furthermore, cycloheximide (CHX) chase assays demonstrated that TTC36 overexpression significantly slowed YBX3 protein degradation (Fig. 5D). Collectively, these results identify TTC36 as a protective factor that stabilizes YBX3 protein.

Next, we directly assessed the ubiquitination levels of YBX3 in Hep3B and SK-HEP-1 cells co-transfected with Flag-tagged YBX3 and HA-tagged ubiquitin. We found that overexpression of TTC36 reduced the ubiquitination level of YBX3, whereas knockdown of TTC36 promoted YBX3 ubiquitination (Fig. 5E). Based on these results, we conclude that TTC36 enhances the stability of YBX3 by binding to it and inhibiting its ubiquitination.

Previous co-immunoprecipitation experiments using truncation mutants revealed that YBX3 interacts with TTC36 via its ARMs/AcidMs domain. We next sought to determine which domain of YBX3 is primarily affected by TTC36 in terms of ubiquitination. Therefore, we examined the effect of TTC36 overexpression on the ubiquitination levels of full-length wild-type YBX3 and truncation mutants #1, #2, and #3. The results showed that overexpression of TTC36 significantly reduced the ubiquitination levels of full-length YBX3 and truncation mutants #1 and #2. In contrast, the ubiquitination level of truncation mutant #3 remained unchanged upon TTC36 overexpression (Fig. 5F). This suggests that TTC36 primarily modulates ubiquitination within the ARMs/AcidMs domain of YBX3.

Subsequently, to identify the specific ubiquitination sites within this domain, we utilized the online tool PhosphoSitePlus (https://www.phosphosite.org/) to predict candidate lysine residues. Four potential sites were identified: K268, K311, K350, and K353 (Fig. 5G). We then constructed YBX3 mutant plasmids and performed ubiquitination assays. The results revealed that mutations at K311 and K350, but not K268 and K353, significantly attenuated YBX3 ubiquitination, indicating that K311 and K350 are critical sites mediating YBX3 ubiquitination (Fig. 5H). Notably, K311 and K350 reside within a region dense with hydrogen bonds mediating the TTC36-YBX3 interaction.

Collectively, these findings demonstrate that TTC36 binds to the ARMs/AcidMs domain of YBX3 and impedes ubiquitination specifically at the K311 and K350 residues, thereby reducing the degradation of YBX3.

### YBX3 Stabilizes SPRED1 mRNA through Direct Binding to Its 3'UTR

**Figure 6:**
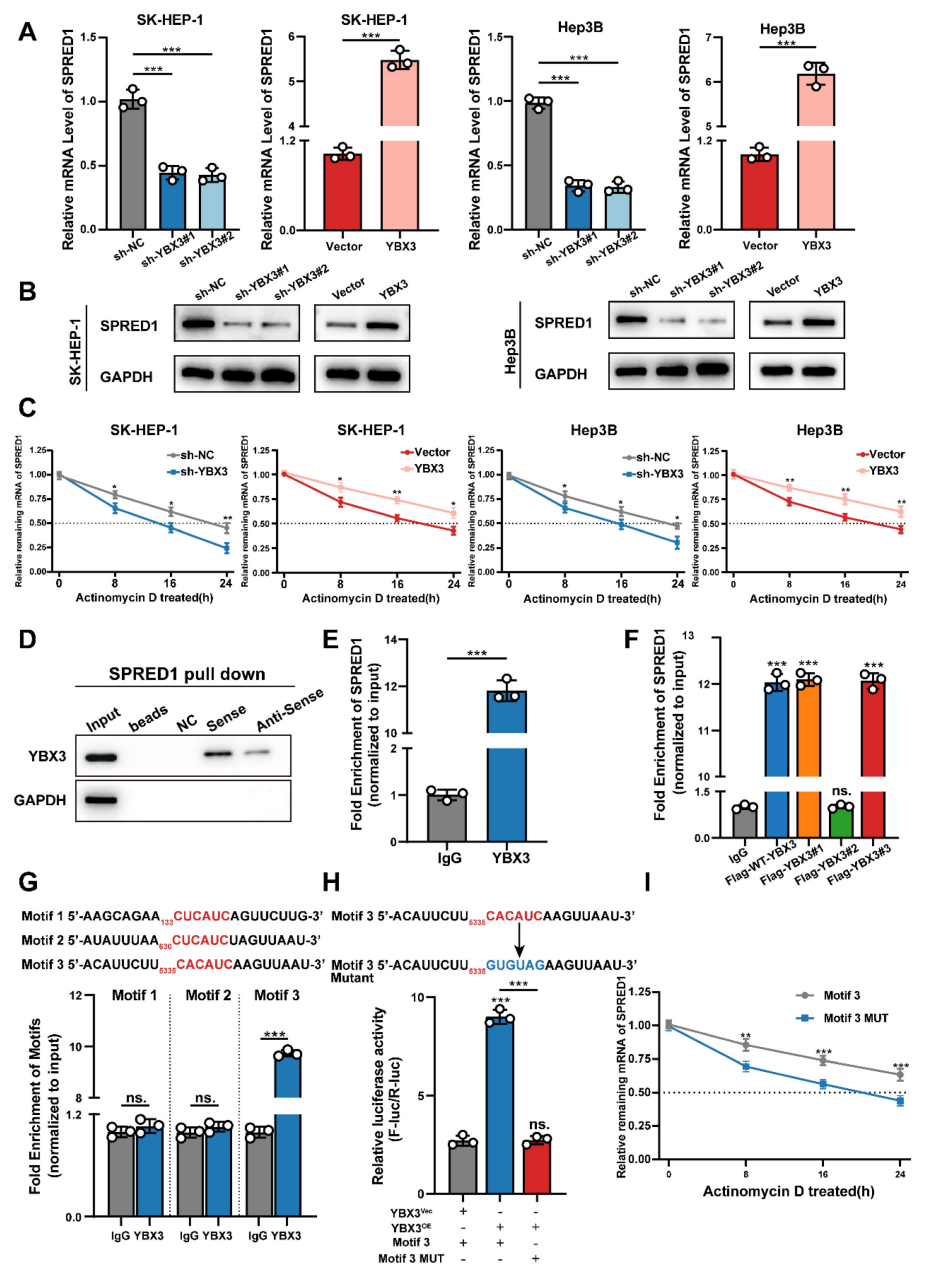
YBX3 stabilizes SPRED1 mRNA through direct 3'UTR binding. **A-B.** SPRED1 mRNA **(A)** and protein **(B)** levels in YBX3-modulated SK-HEP-1 and Hep3B cells. **C.** mRNA stability assays with actinomycin D. **D.** Western blotting analysis of RNA-pulldown products using biotinylated SPRED1 sense or antisense probes. **E.** RIP-qPCR showing SPRED1 mRNA enrichment with YBX3 antibody. **F.** RIP-qPCR of SPRED1 mRNA binding to YBX3 truncation mutants with FLAG antibody. **G.** Motif-specific RIP-qPCR of SPRED1 3'UTR fragments. **H.** Luciferase activity of wild-type or mutant Motif 3 reporters with YBX3 overexpression. **I.** mRNA stability assays comparing wild-type/mutant Motif 3 constructs.

​Previous studies have demonstrated that YBX3 promotes mRNA stability to enhance protein expression 15,16,23,24. We therefore investigated whether TTC36 upregulates SPRED1 expression by facilitating YBX3-mediated stabilization of SPRED1 mRNA. First, we assessed the effect of YBX3 on SPRED1 mRNA and protein levels in Hep3B and SK-HEP-1 cell lines. Results showed that YBX3 overexpression increased both SPRED1 mRNA and protein levels, whereas sh-YBX3 reduced SPRED1 expression (Fig. 6A-B). We then measured the impact of YBX3 on SPRED1 mRNA half-life. YBX3 overexpression significantly inhibited SPRED1 mRNA degradation, while sh-YBX3 shortened its half-life (Fig. 6C). These findings indicate that YBX3 upregulates SPRED1 expression by stabilizing its mRNA.

To validate the direct binding between YBX3 and SPRED1 mRNA, we performed RNA pulldown assays using biotinylated sense and antisense transcripts of SPRED1. Analysis of pulldown products revealed significantly greater enrichment of YBX3 with the sense strand (Fig. 6D). RNA immunoprecipitation followed by qPCR (RIP-qPCR) using a YBX3 antibody further confirmed this interaction (Fig. 6E).

Given that Y-box family proteins recognize nucleotide sequences via their highly conserved cold shock domain (CSD) 25-27, we investigated whether YBX3 binding to SPRED1 mRNA requires this domain. RIP-qPCR using Flag-tagged YBX3 truncation mutants demonstrated that deletion of the CSD (Flag-YBX3#2) markedly reduced SPRED1 mRNA enrichment (Fig. 6F). Together, these results establish that YBX3 binds SPRED1 mRNA through its CSD domain to promote mRNA stability and enhance protein expression.

Previous studies indicate that YBX3 enhances mRNA stability through 3'UTR binding 15 via its CSD domain recognizing the C(N)CAUC motif 25,28-31, we therefore scanned the 3'UTR of SPRED1 mRNA and identified three candidate binding motifs: Motif 1 (133-138 nt: CUCAUC), Motif 2 (630-635 nt: CUCAUC), and Motif 3 (5335-5340 nt: CACAUC). Expression plasmids containing each candidate sequence flanked by 200-bp upstream/downstream regions were co-transfected with YBX3 plasmid into HEK293T cells. RIP-qPCR revealed that YBX3 bound significantly to Motif 3 compared to IgG control, while Motif 1 and 2 showed no significant enrichment (Fig. 6G). We subsequently inserted the 3'UTR containing wild-type Motif 3 or its mutant (CACAUC→GUGUAG) to the downstream of a firefly luciferase reporter. Following co-transfection with YBX3 and Renilla luciferase control plasmids into HEK293T cells, luciferase activity analysis demonstrated that YBX3 overexpression significantly enhanced luciferase activity for the wild-type Motif 3, but not for the mutant sequence (Fig. 6H). Furthermore, mutation of Motif 3 significantly shortened the half-life of SPRED1 mRNA (Fig. 6I).

Collectively, these data demonstrate that YBX3 recognizes and binds the CACAUC motif within the 3'UTR of SPRED1 mRNA via its CSD domain. This interaction enhances mRNA stability and promotes SPRED1 protein expression.

### TTC36 Regulates HCC Proliferation through the YBX3/SPRED1/Ras/MAPK Axis

Our earlier work established TTC36 as a regulator of SPRED1 via YBX3-mediated mRNA stabilization. To validate the functional significance of this axis, we performed systematic rescue experiments across *in vitro* and *in vivo*.

**Figure 7:**
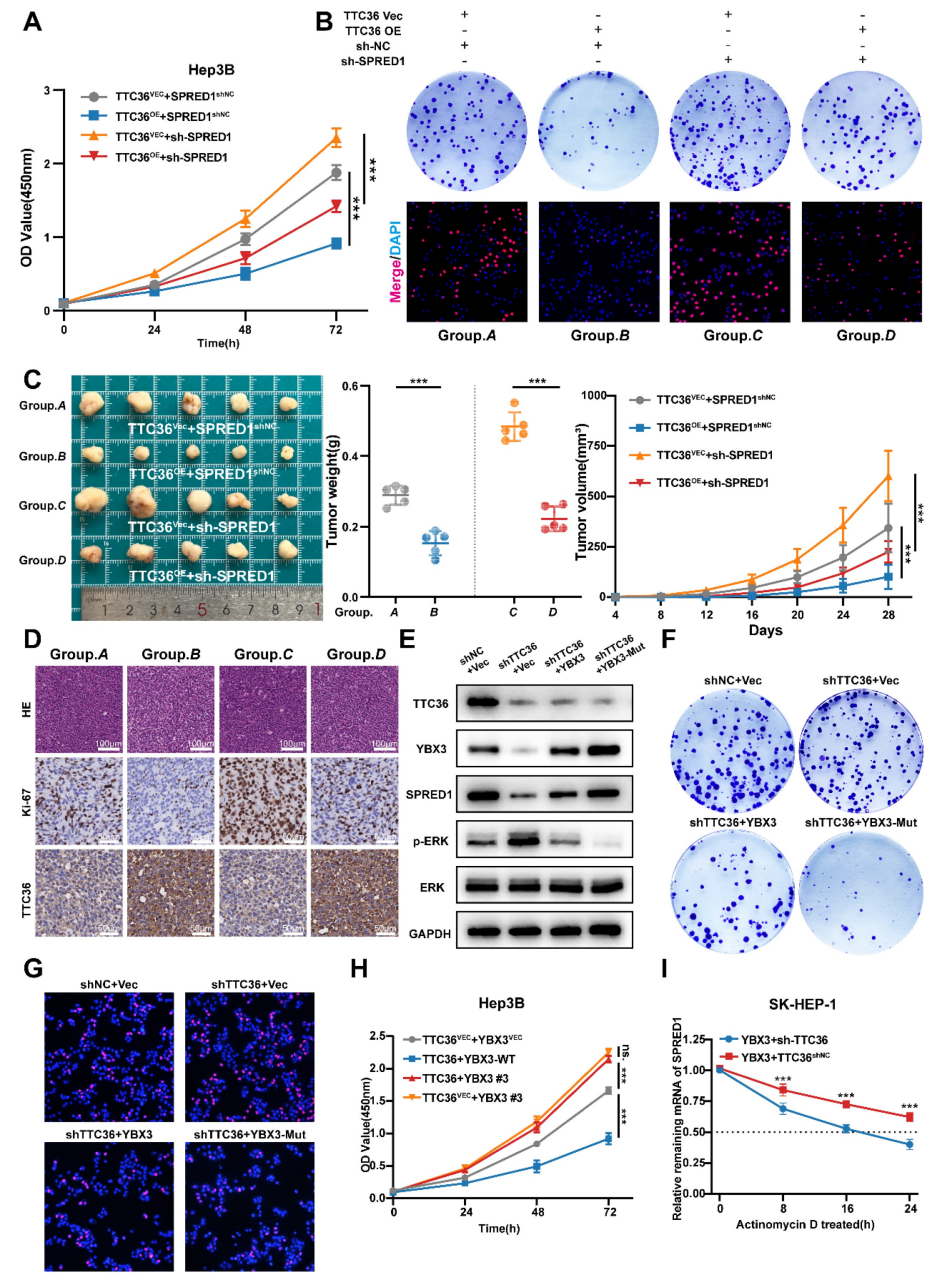
Functional validation of the TTC36/YBX3/SPRED1/Ras/MAPK axis. **A-B.** Functional assays: CCK-8 proliferation, colony formation, and EdU incorporation in TTC36 and SPRED1-modulated Hep3B cells. **C.** Image of xenograft tumors and growth weights (left), volumes (right) from four Hep3B groups. **D.** HE and IHC staining (Ki-67 and TTC36) of xenograft tumors. **E.** western blot analysis of TTC36, YBX3, SPRED1, p-ERK1/2 and ERK in SK-HEP-1 cells transfected with shTTC36 and YBX3/YBX3-Mutant. **F.** colony formation assay using SK-HEP-1 cells transfected with shTTC36 and YBX3/YBX3-Mutant**. G.** Edu staining assay using SK-HEP-1 cells transfected with shTTC36 and YBX3/YBX3-Mutant. **H.** CCK-8 assay of cells expressing TTC36 and YBX3-WT/Flag-YBX3#3. **I.** SPRED1 mRNA stability in YBX3-overexpressing with/without TTC36 knockdown.

*In vitro* experiments demonstrated that TTC36 overexpression upregulated SPRED1 mRNA levels. This effect was attenuated by co-expression of sh-SPRED1 and enhanced by SPRED1 overexpression plasmid, respectively Fig. S5A). We subsequently assessed the activation status of the Ras/MAPK pathway under these conditions. TTC36 overexpression significantly reduced the phosphorylation levels of ERK1/2. However, this suppression was partially reversed by sh-SPRED1-mediated SPRED1 knockdown. Conversely, introducing the SPRED1 overexpression plasmid further decreased ERK1/2 phosphorylation in TTC36-overexpressing cells (Fig. S5B). These results indicate that TTC36 regulates Ras/MAPK signaling activation by modulating SPRED1 expression. Consistently, TTC36-mediated proliferation inhibition (CCK-8, colony formation, EdU) was ​fully reversed by SPRED1 knockdown (Fig. 7A-B, Fig.S5C), collectively confirming SPRED1's indispensable role in TTC36's anti-proliferative action.

*In vivo*, subcutaneous xenografts recapitulated the TTC36-SPRED1 regulatory axis: ​TTC36 overexpression significantly inhibited tumor growth. Co-transfection of sh-SPRED1 partially reversed this suppressive effect. (Fig. 7C). Consistently, immunohistochemical (IHC) analysis of Ki67 staining revealed that TTC36 overexpression reduced Ki67 expression, while SPRED1 knockdown substantially increased Ki67 expression. Importantly, sh-SPRED1 rescued the pronounced decrease in Ki67 expression induced by TTC36 overexpression. (Fig. 7D, Fig.S5D). These results confirmed an interaction where TTC36 upregulates SPRED1 to constrain HCC growth.

Next, to genetically validate the functional dependency of TTC36 on YBX3 stabilization, we expressed the ubiquitination-resistant mutant YBX3-K311/350R in TTC36-knockdown SK-HEP-1 cells. Notably, YBX3-Mut rescued SPRED1 protein levels, suppressed ERK hyperphosphorylation (Fig. 7E), and reversed the accelerated proliferation induced by TTC36 deficiency (Fig. 7F-G, Fig. S5E-F). These results indicate that the anti-tumor effects of TTC36 require the integrity of YBX3's K311/K350 residues, directly linking TTC36-mediated ubiquitination shielding to YBX3-SPRED1 axis activation.

In addition, disrupting the interaction between TTC36 and YBX3 via deleting the binding region ARMs/AcidMs domain of YBX3 reversed TTC36's anti-proliferation effect (Fig. 7H). Moreover, TTC36 knockdown accelerated SPRED1 mRNA degradation even with YBX3 overexpression (Fig. 7I), definitively positioning TTC36 as essential for YBX3-mediated mRNA-stabilization.

To sum up, our data delineate the ​TTC36/YBX3/SPRED1/Ras/MAPK axis as a ​core regulatory machinery restraining HCC proliferation.

### TTC36 Expression Modulates HCC Sensitivity to Sorafenib

Sorafenib, a first-line multi-kinase inhibitor for HCC, primarily targets the Ras/MAPK pathway by inhibiting B-Raf and C-Raf. Building on our findings that TTC36 constrains HCC proliferation via suppressing Ras/MAPK signaling, we hypothesized that high TTC36 level may force tumor cells to rely on alternative proliferation-promoting pathways, thereby inducing resistance to sorafenib. Conversely, low TTC36 expression retains Ras/MAPK dependency, sensitizing HCC to sorafenib. To test this, we evaluated sorafenib sensitivity across HCC *in vitro* and* in vivo* models with TTC36 modulated. Firstly, Hep3B and SK-HEP-1 cells were treated with sorafenib at increasing concentrations (0.5 µM, 1 µM, 2 µM, 5 µM, 10 µM, and 20 µM) for 48 hours, and cell viability curves were generated. As shown in Fig. S6A, TTC36-overexpressing Hep3B cells exhibited an IC₅₀ of 5.39 ± 0.75 µM, significantly higher than that of empty vector control cells (3.54 ± 0.75 µM). Similarly, in SK-HEP-1 cells, TTC36 knockdown significantly enhanced sorafenib's cytotoxic effect, yielding an IC₅₀ of 3.42 ± 0.77 µM - significantly lower than the control group (8.08 ± 0.65 µM). Additionally, sorafenib-resistant Hep3B (Hep3B SR) and SK-HEP-1 (SK-HEP-1 SR) cell lines were obtained and characterized. IC₅₀ values confirmed their resistant phenotype compared to parental cells (Hep3B P and SK-HEP-1 P) (Fig. S6B). Subsequent qPCR and Western blot analyses revealed significantly elevated TTC36 expression levels in the resistant cell lines relative to their parental counterparts (Fig. S6C). These results demonstrate that TTC36 expression influences sorafenib sensitivity in HCC cells.

**Figure 8:**
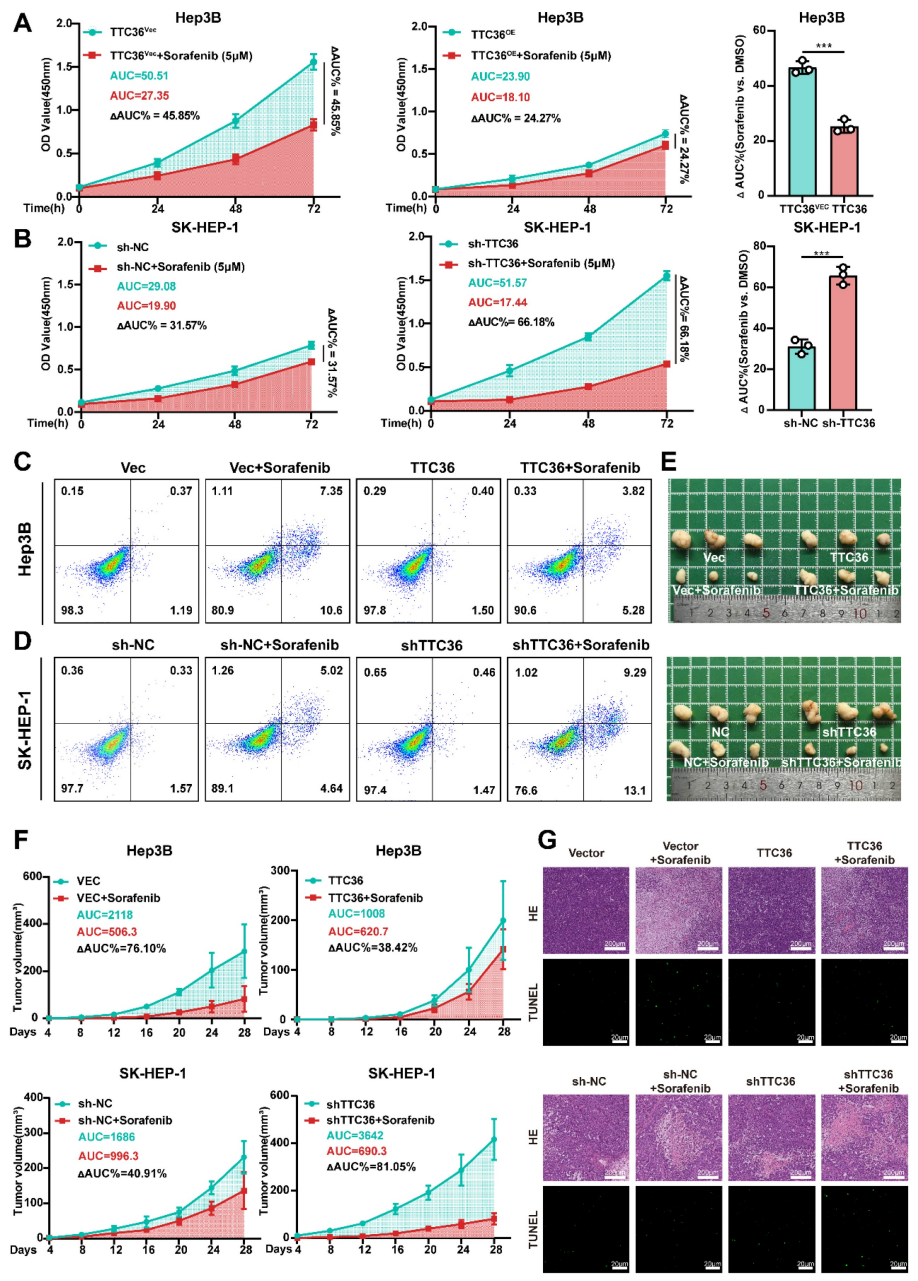
TTC36 expression inversely correlates with sorafenib sensitivity. **A-B.** Sorafenib response in **(A)** TTC36-vector or -overexpressing Hep3B and **(B)** TTC36 control or knockdown SK-HEP-1 cells. AUC quantification of proliferation curves. All cells were treated with 5µM Sorafenib for 48h. **C-D.** Apoptosis analysis of TTC36-modified Hep3B and SK-HEP-1 cells treated with 5µM Sorafenib for 48h. **E.** Image of xenograft tumors with or without sorafenib treatment (Mice was treated with Sorafenib 4mg/kg/d via gavage administration for 4 weeks). **F.** Tumor growth curves and AUC quantification post-sorafenib treatment. **G.** HE staining (necrosis areas) and TUNEL staining (apoptosis) in xenografts.

Next, to further confirm the function of TTC36 in sorafenib resistance, we detected cell viability after sorafenib (5µM) treatment for 48h, and area under the curve (ΔAUC) was calculated. We found that relatively low level of TTC36 in HCC cells caused significant proliferation inhibitory effects but had less of an impact on TTC36-high expressed HCC cells, which further confirmed that TTC36's role in the maintenance of sorafenib resistance (Fig. 8A-B). Apoptosis analysis demonstrated that ​TTC36 overexpression suppressed sorafenib-induced apoptosis in Hep3B cells (Fig. 8C), ​whereas TTC36 knockdown sensitized SK-HEP-1 cells to sorafenib-triggered apoptosis (Fig. 8D). These *in vitro* findings were mirrored *in vivo*: TTC36-overexpressing xenografts showed diminished sorafenib response (ΔAUC%: 38.42% vs. 76.10% in controls), while TTC36-knockdown tumors exhibited enhanced sensitivity (ΔAUC%: 81.05% vs. 40.91% in controls), highlighting a clear TTC36-dependent therapeutic disparity (Fig. 8E-F). Histopathology and TUNEL staining ​further validated these findings: TTC36 overexpression ​attenuated sorafenib-induced necrosis and apoptosis, ​whereas knockdown enhanced both responses to sorafenib treatment (Fig. 8G).

Collectively, our data demonstrate that TTC36 plays a ​key role in HCC resistance to sorafenib.

### TTC36 Drives Sorafenib Resistance via Compensatory PI3K/Akt Activation

Compensatory activation of pro-proliferative signaling pathways constitutes a major mechanism underlying acquired resistance in hepatocellular carcinoma (HCC). Both PI3K/Akt and JAK/STAT signaling pathways have been implicated in HCC progression and therapeutic resistance (32,33. However, whether these pathways undergo compensatory activation to sustain tumor cell growth when Ras/MAPK signaling is suppressed by TTC36 required further investigation. To address this, Hep3B cells transfected with TTC36 expression plasmid or empty vector were serum-starved in 1% FBS medium for 12 hours, followed by stimulation with either the PI3K/Akt agonist EGF (50 ng/mL) or the JAK/STAT agonist IL-6 (50 ng/mL) for 20 minutes. Cells were immediately lysed for protein analysis. Both pathways exhibited low basal phosphorylation levels after starvation, which were unaffected by TTC36 overexpression. Upon EGF stimulation, TTC36-overexpressing cells showed significantly enhanced PI3K/Akt pathway activation, as evidenced by markedly increased phosphorylation levels. In contrast, while IL-6 effectively induced JAK and STAT3 phosphorylation, TTC36 overexpression did not potentiate this activation. These findings demonstrate that TTC36 specifically enables compensatory hyperactivation of the PI3K/Akt pathway in response to growth factor stimulation, providing a potential mechanism for TTC36-induced sorafenib resistance in HCC.

**Figure 9:**
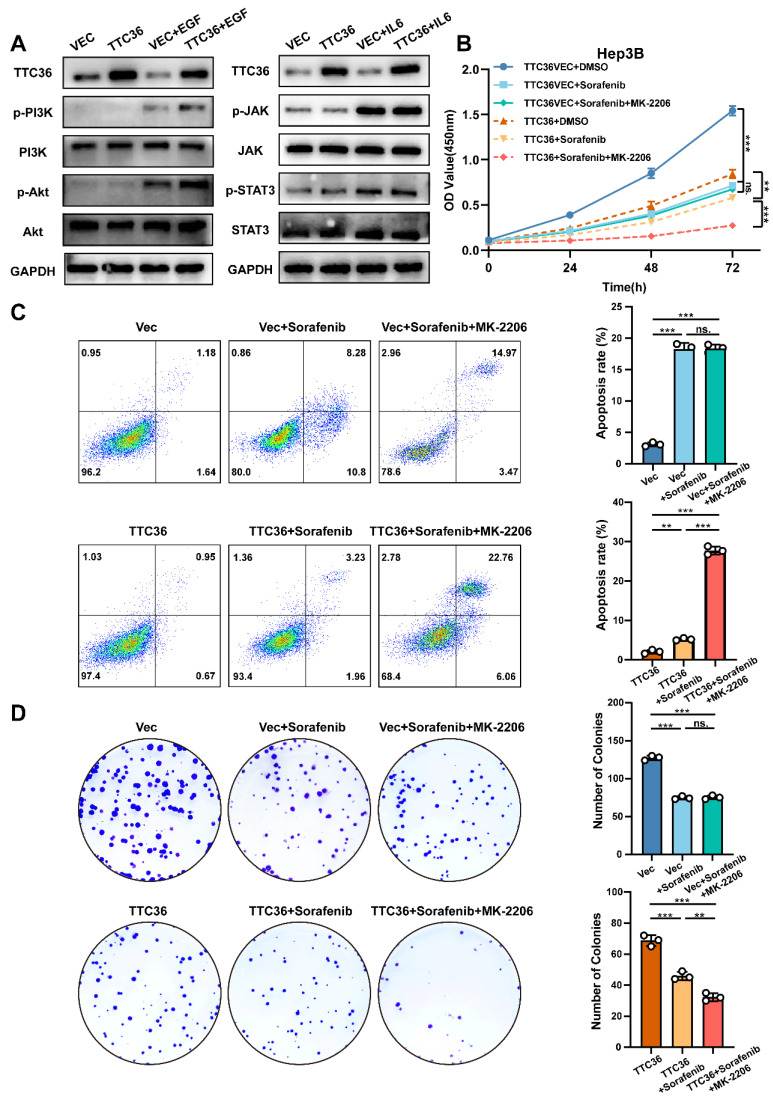
TTC36 induces sorafenib resistance via compensatory PI3K/Akt activation. A. Western blot analysis of PI3K/Akt signaling and JAK/STAT signaling activation in TTC36-mediated Hep3B cells treated with EGF (50ng/mL) or IL-6 (50ng/mL), respectively. Cells were cultured in 1% FBS DMEM for 12 hours before adding EGF and IL-6. B. CCK-8 assay using TTC36-mediated Hep3B cells treated with DMSO (10%), sorafenib (5µM) or sorafenib (5µM) +MK-2206 (2µM) for 48h. C-D. Apoptosis analysis and colony formation assay of TTC36-mediated Hep3B cells treated with DMSO (10%), sorafenib (5µM) or sorafenib (5µM) +MK-2206 (2µM) for 48h.

To further confirm the role of PI3K-Akt activated in inducing sorafenib resistance under this situation, we combined Akt inhibitor (MK-2206) with sorafenib in TTC36-OE cells. Strikingly, this combination synergistically enhanced apoptosis and suppressed cell viability and colony formation (Fig. 9B-D), effectively reversing the compensatory resistance. This confirms that PI3K/Akt hyperactivation is an actionable target to restore sorafenib efficacy in TTC36-high HCC.

Taken together, we conclude that compensatory activation of PI3K/Akt signaling when TTC36 is highly expressed is the crucial factor that induces sorafenib resistance in HCC cells.

## Discussion

This study establishes TTC36 as a pivotal tumor suppressor in hepatocellular carcinoma that orchestrates a previously unrecognized TTC36/YBX3/SPRED1 signaling axis which suppresses Ras/MAPK pathway to constrain tumor proliferation. We demonstrate that TTC36 downregulation is a consistent feature of HCC, correlating with aggressive progression and poor prognosis. Mechanistically, TTC36 directly binds and stabilizes the RNA-binding protein YBX3 by physically masking its key ubiquitination sites (K311 and K350), thereby preventing proteasomal degradation. Stabilized YBX3 in turn enhances the mRNA stability of SPRED1 to inhibit ERK1/2, but not p38 or JNK activation through direct interaction with the CACAUC motif in its 3'UTR, ultimately inhibiting Ras/MAPK signaling and HCC proliferation. Notably, while this axis suppresses tumor growth, it simultaneously induces compensatory PI3K/Akt hyperactivation—a paradoxical adaptation that confers resistance to sorafenib. Critically, this resistance can be reversed by co-targeting Akt, revealing a rational combination strategy for TTC36-high HCC.

In our study, we confirmed that TTC36 is downregulated and associated with poor prognosis in HCC, which is consistent with the conclusions of previous studies 5,6,8. Interestingly, Xie et al. (2025) demonstrate its oncogenic role through c-Myc stabilization 34. This apparent contradiction may reflect cellular context-dependency: TTC36 likely exerts distinct functions based on dominant signaling pathways (Ras vs. c-Myc) and post-translational modifications (e.g., S125 phosphorylation). Future studies should investigate whether TTC36 expression patterns, phosphorylation status, and pathway activation profiles can stratify HCC patients for targeted therapies.

In addition, our work significantly advances the understanding of post-transcriptional regulation in HCC pathogenesis. While SPRED1 is reported to be downregulated via promoter methylation or miRNA interaction 12-14, we uncover a novel layer of regulation wherein TTC36 governs SPRED1 mRNA stability via YBX3 stabilization. This represents a rare example of a tumor suppressor modulating RNA-binding protein function through ubiquitination shielding, extending beyond classical transcriptional or epigenetic mechanisms. The identification of K311/K350 as critical ubiquitination sites within YBX3's ARMs/AcidMs domain—a region mediating TTC36 binding—provides new insight into how protein-protein interactions regulate RBP stability. Importantly, genetic rescue experiments confirmed that both the TTC36-YBX3 interaction and YBX3-mediated SPRED1 stabilization are indispensable for TTC36's anti-proliferative function, solidifying the axis as a core regulatory machinery in HCC. Notably, Xie. et al. (2019) discovered that TTC36 participates in post-translational modification regulation by suppressing HPD polyubiquitination, though the specific mechanism remained unclear 21. Our results expand our understanding of TTC36-mediated ubiquitination regulation, providing structural biology insights into TPR proteins.

The most intriguing and clinically relevant finding of our study is the paradoxical role of TTC36 in conferring sorafenib resistance. While it robustly suppresses tumor growth by inhibiting the Ras/MAPK pathway, it simultaneously renders HCC cells less sensitive to sorafenib, a multi-kinase inhibitor that primarily targets this same pathway. This apparent contradiction can be explained by the concept of 'oncogenic bypass' or 'adaptive resistance' 35. In TTC36-high tumors, the constitutive suppression of Ras/MAPK signaling creates a selective pressure for cancer cells to exploit alternative survival pathways. As we demonstrated, the PI3K/Akt pathway is hyperactivated as a compensatory mechanism, thereby sustaining cell proliferation and diminishing the dependency on the Ras/MAPK axis that sorafenib aims to block. This phenomenon mirrors the context-dependent roles of other well-established tumor suppressors, such as p53, which can also modulate drug sensitivity in opposing ways depending on the genetic background 9,10.

Rather than diminishing its value, this dual nature of TTC36 positions it as a powerful predictive biomarker for personalized medicine in HCC. Our data propose a novel stratification strategy: patients with TTC36-low tumors, which exhibit Ras/MAPK dependency, are likely to be optimal candidates for sorafenib monotherapy. Conversely, patients with TTC36-high tumors could be preemptively directed towards combination therapies that co-target the compensatory PI3K/Akt pathway, such as sorafenib plus an Akt inhibitor (e.g., MK-2206), to overcome intrinsic resistance. This approach could significantly improve the efficacy of first-line treatment and avoid the futile administration of sorafenib to a subset of patients who are unlikely to benefit.

Limitations of this study include unexplored roles of TTC36 in immune modulation, and the need to validate this axis in *in situ* HCC models. Future work should investigate whether TTC36 regulate the immune microenvironment and more clinical data of HCC patients who undergo immune therapies should be collected to explore whether TTC36 loss has an impact on immune therapy response in HCC patients.

In conclusion, our study elucidates a novel TTC36/YBX3/SPRED1/Ras/MAPK regulatory axis in HCC, highlighting TTC36 as a promising therapeutic target and prognostic marker. The dual role of TTC36 in tumor suppression and sorafenib sensitivity underscores the need for personalized treatment strategies in HCC, paving the way for future clinical applications.

## Conclusion

In summary, our findings demonstrate that TTC36 functions as a tumor suppressor in HCC by inhibiting the Ras/MAPK signaling pathway via YBX3/SPRED1 axis. Furthermore, TTC36 modulates sorafenib sensitivity via compensatory PI3K/Akt activation, with high TTC36 expression conferring resistance and low expression enhancing sensitivity. These results illustrate how molecular dissection of tumor suppressor networks can reveal unexpected therapeutic liabilities, providing a foundation for the development of novel therapeutic strategies for HCC.

## Supplementary Material

Supplementary figures.

Supplementary tables.
