# Supplementary material for: TTC36-Mediated Tumor Suppression via YBX3/SPRED1 Axis Paradoxically Reduces Sorafenib Sensitivity in Hepatocellular Carcinoma
Source: Int J Biol Sci. 2025 Oct 1;21(14):6351–72. doi: 10.7150/ijbs.115727 (PMC12594589; doi:10.7150/ijbs.115727)
Supplement: Supplementary file 1 — Supplementary figures. [file ijbsv21p6351s1.pdf]

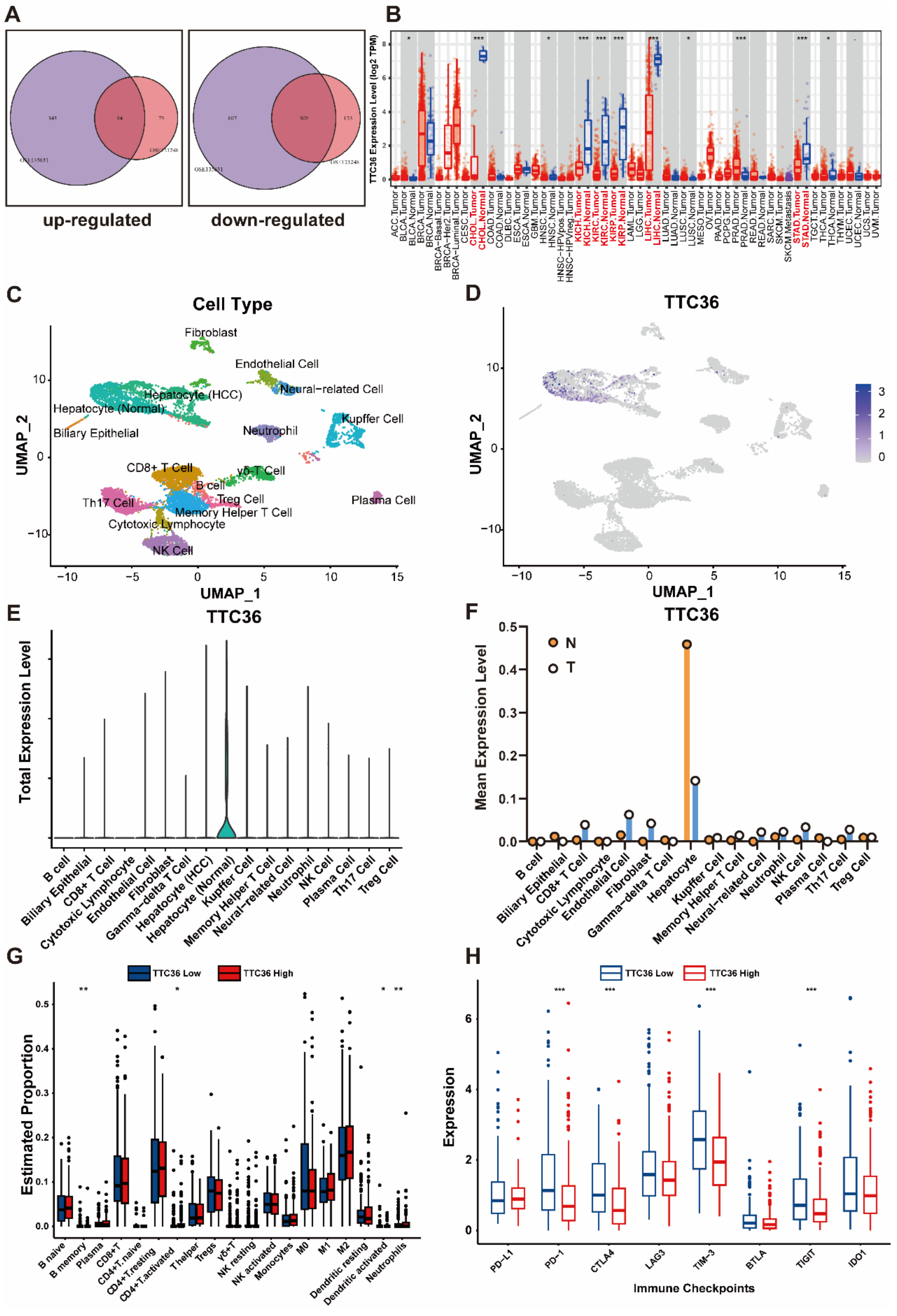

Figure S1. Expression analysis of TTC36 in RNA-seq and scRNA-seq data. **A.** Cross-dataset intersection of DEGs from GSE121248 and GSE135631. **B.** pan-cancer analysis

of TTC36 expression based on TCGA database (malignant categories with significant TTC36 down-regulation are marked in red fonts). **C.** Analysis of scRNA-seq data for GSE282701: Cell dimensionality reduction clustering and cell annotation results. **D.** Analysis of TTC36 Expression Levels Across Cell Clusters. **E.** Violin Plot of TTC36 Expression Level Across Cell Clusters. **F.** Comparative analysis of mean TTC36 expression in tumor and matched non-tumor tissues across major cell clusters. **G.** Analysis of immune cell infiltration in HCC stratified by TTC36 expression levels. **H.** Expression profiles of key immune checkpoint molecules in HCC stratified by TTC36 expression levels.

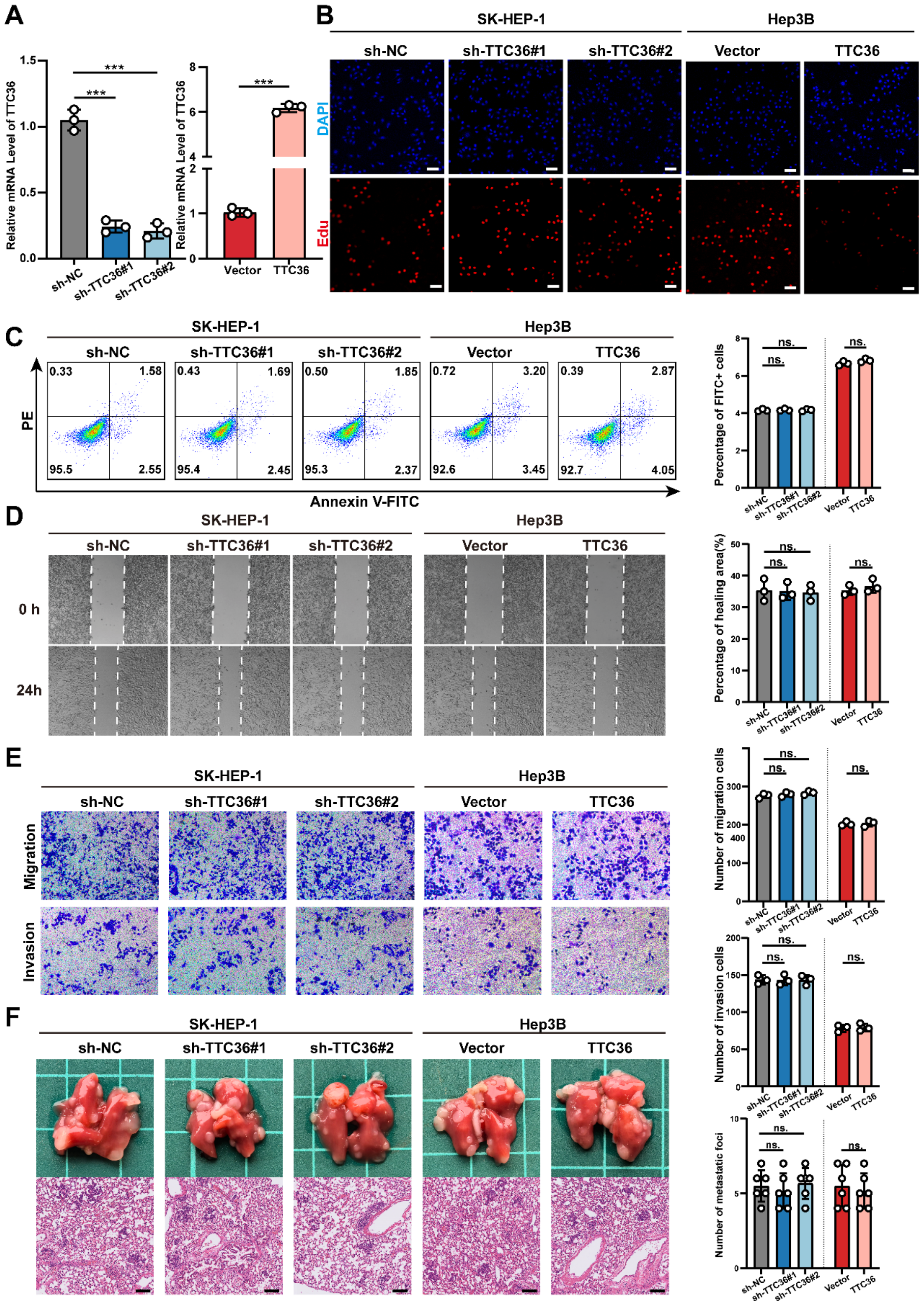

Figure S2. Functional validation of TTC36 *in vivo* and *in vitro* models. **A.** qRT-PCR analysis of TTC36 knockdown and overexpression efficiency in SK-HEP-1 cells and Hep3B cells. **B.** Images of EdU incorporation assays in TTC36-modulated cells (blue: DAPI; red: EdU. Merge images are displayed in Figure 2). **C.** Apoptosis analysis of

TTC36-modified SK-HEP-1 and Hep3B cells. **D.** Wound healing assays showing the migratory capacity of TTC36-modulated cells. Wound closure rates were quantified at 24h post-scratch. **E.** Transwell migration and invasion assays demonstrating the impact of TTC36 knockdown and overexpression on cellular migration or invasiveness. **F.** *In vivo* lung metastasis model constructed via tail injection using TTC36-modified SK-HEP-1 and Hep3B cells. Scale bar: 100μm.

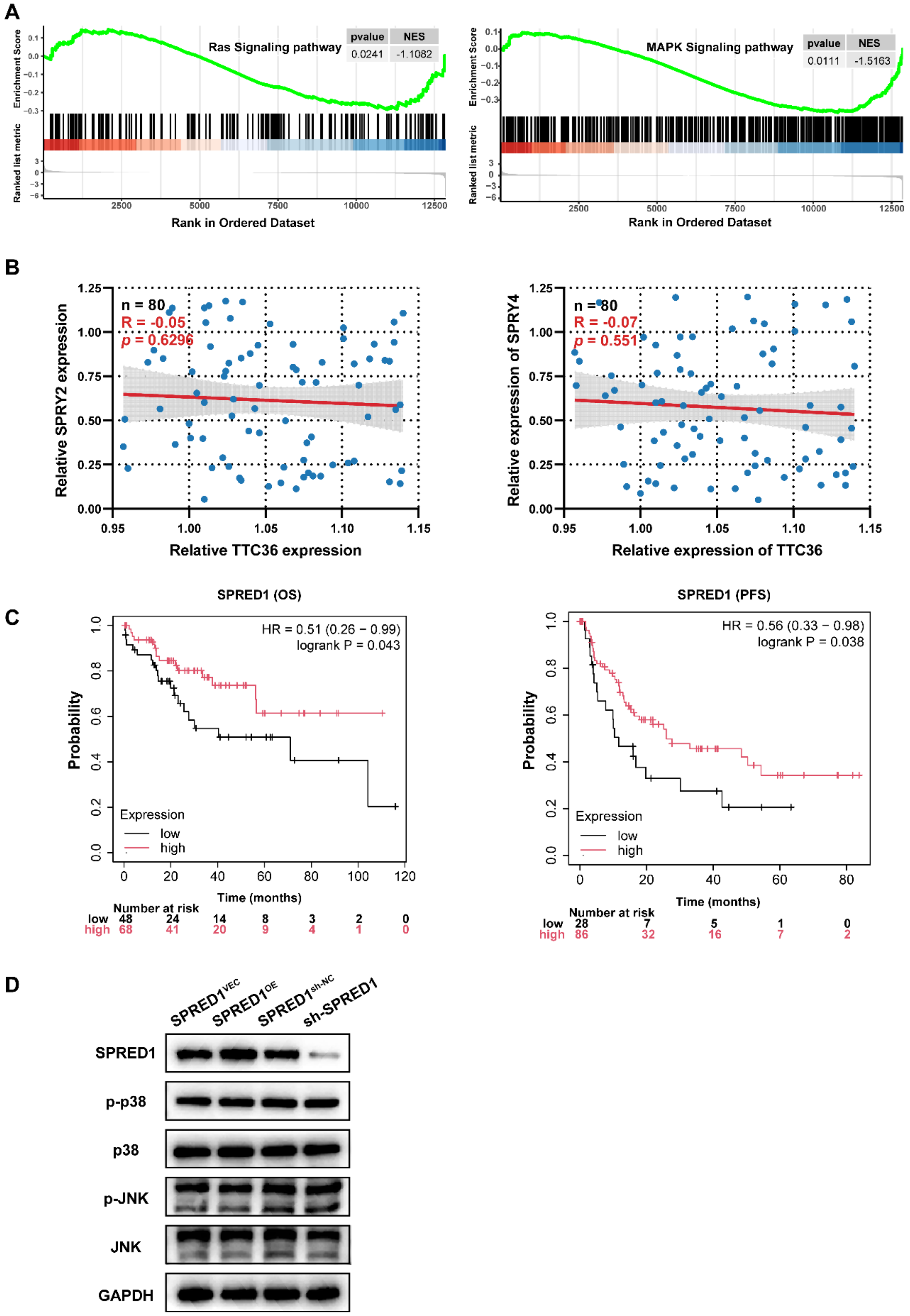

Figure S3. TTC36 inhibits Ras/MAPK signaling through regulating SPRED1. **A.** GSEA plots showing suppression of Ras and MAPK signaling pathways in TTC36 overexpressed cells. **B.** Correlation analysis of TTC36 with SPRY2 and SPRY4 in

HCC clinical samples (no significant correlation). **C.** Survival analysis of SPRED1 in HCC patients using Kaplan-Meier plotter (high vs. low, OS, *P* = 0.043; PFS, *P* = 0.038)**.** **D.** Detection of phosphorylation levels in Ras/MAPK downstream branches p38 and JNK via western blot.

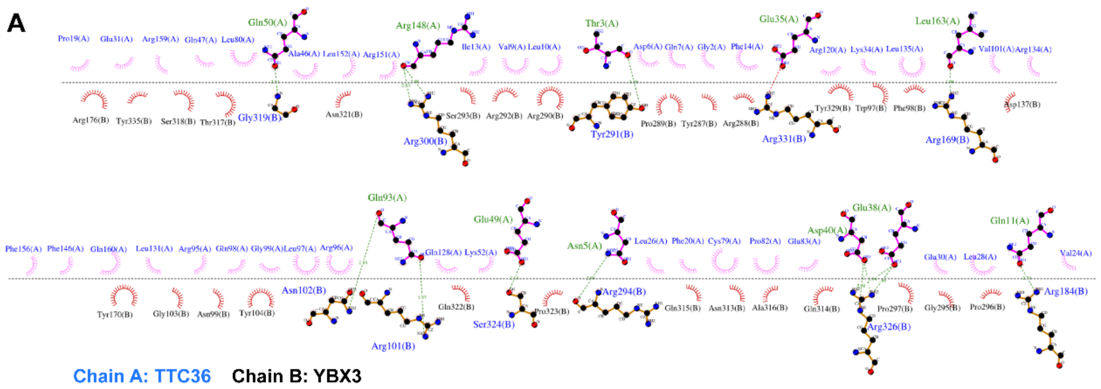

Figure S4. Structural analysis of the TTC36-YBX3 interaction. **A.** Detailed hydrogen bond network at the TTC36-YBX3 interface.

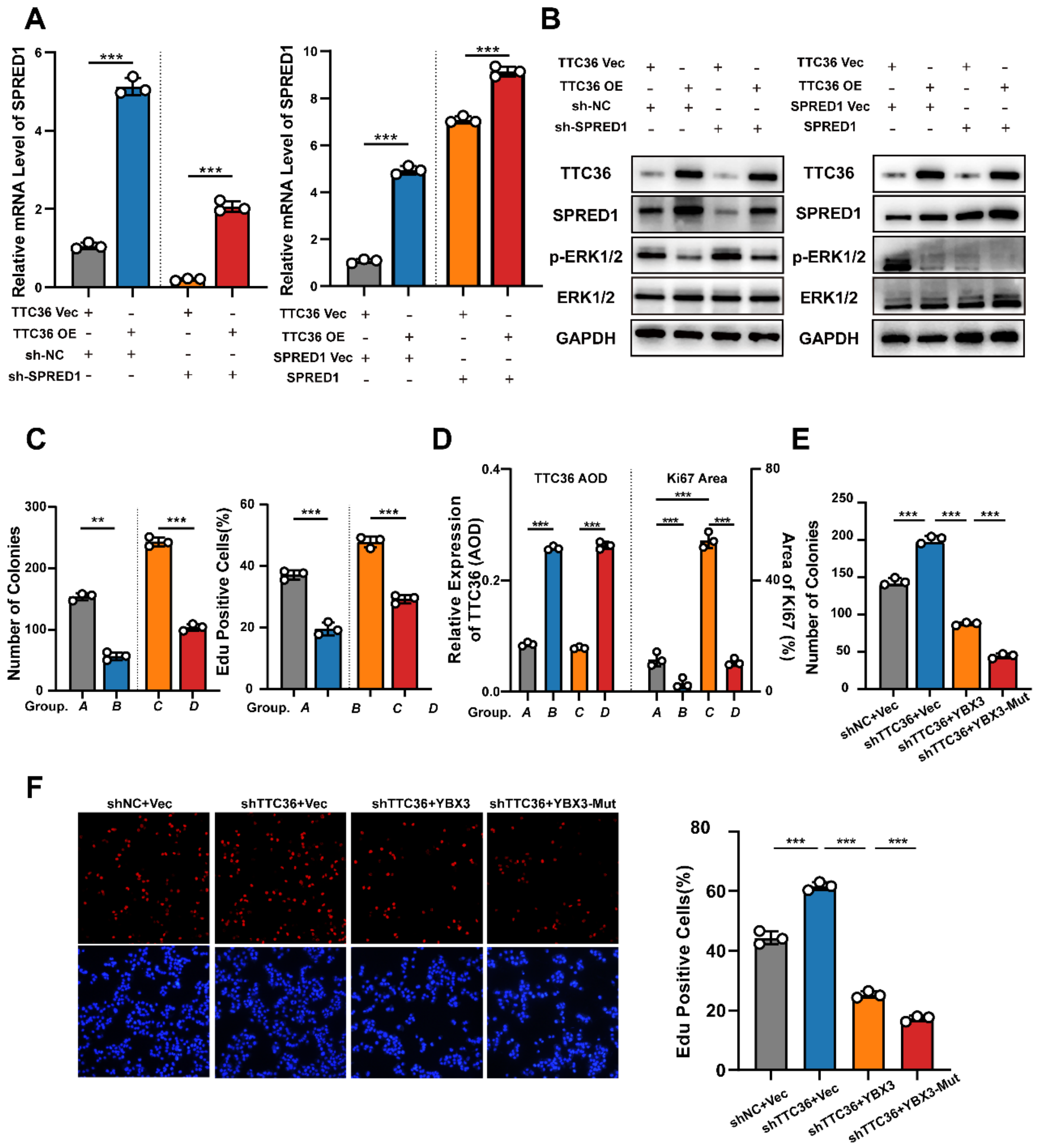

Figure S5. Functional validation of TTC36/YBX3/SPRED1 axis in HCC suppression.

A. qRT-PCR analysis of SPRED1 mRNA in TTC36-modulated Hep3B cells co-transfected with sh-SPRED1 or SPRED1 expression plasmid. **B.** Western blot analysis of SPRED1 expression and ERK1/2 activation level in Hep3B cells co-transfected with TTC36 plasmid and sh-SPRED1/SPRED1 plasmid. **C.** Quantitative statistical analysis of colony formation assay and Edu staining assay displayed in Figure 7B. **D.** Quantitative statistical analysis of TTC36 expression and percentage of Ki67 area in the IHC staining displayed in Figure 7D. **E.** Quantitative statistical analysis of colony formation assay displayed in Figure 7F. **F.** Images and quantitative statistical analysis of EdU staining assay in sh-TTC36-modulated SK-HEP-1 cells co-transfected with

wild type YBX3 or K311/K350 mutant YBX3 (blue: DAPI; red: EdU. Merge images are displayed in Figure 7G)

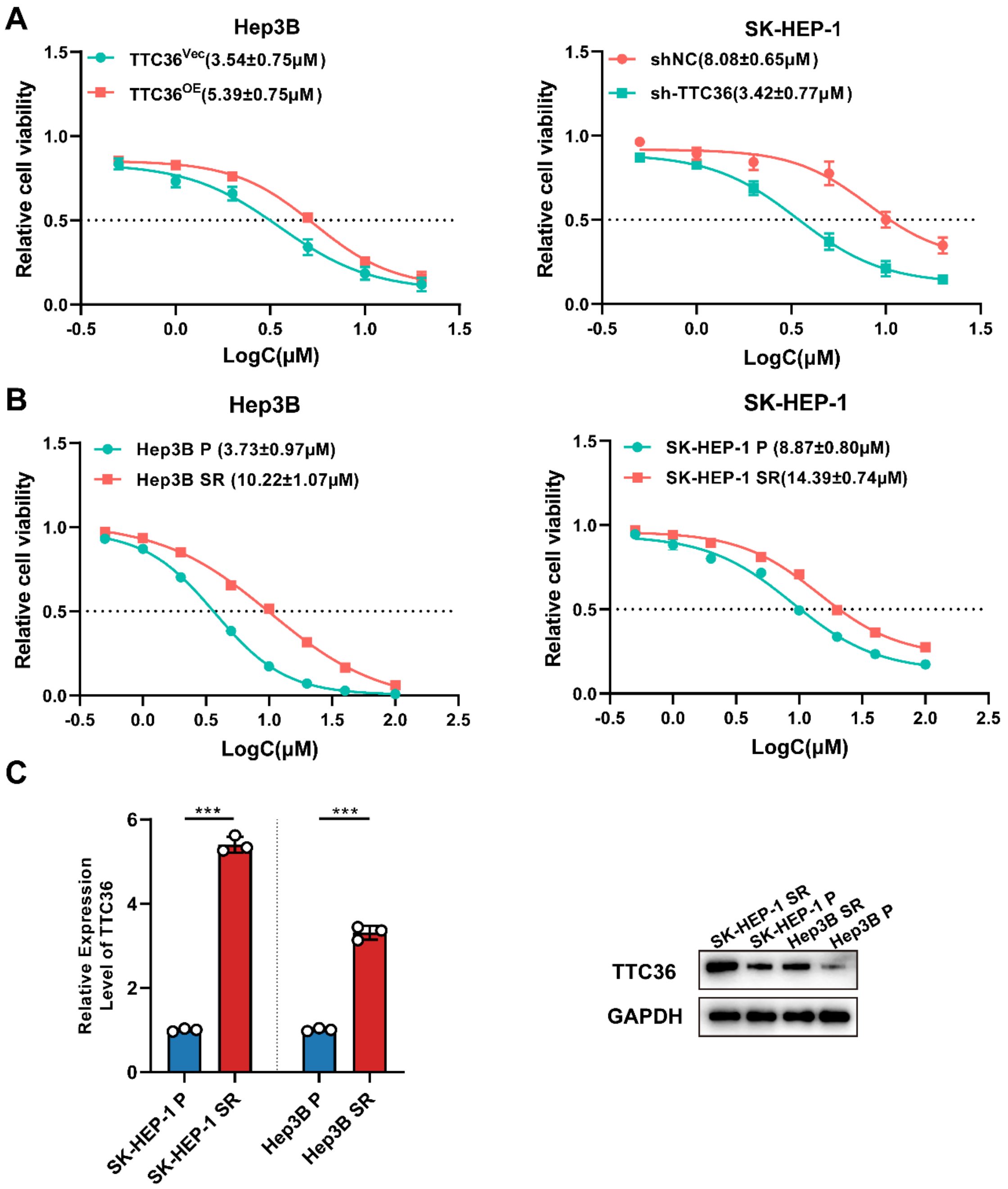

Figure S6. Examination associated with sorafenib resistance in Hep3B and SK-HEP-1 cells.

A. IC50 curves of TTC36-modulated Hep3B and SK-HEP-1 cells treated with sorafenib at the concentration 0.5 μM, 1 μM, 2 μM, 5 μM, 10 μM, and 20 μM for 48 hours. **B.** IC50 curves of sorafenib resistant and parental Hep3B and SK-HEP-1 cells. (Labeled as Hep3B SR, SK-HEP-1 SR, Hep3B P and SK-HEP-1 P, respectively.) C. qRT-PCR

and western blot analysis of TTC36 expression in sorafenib resistant or parental SK-HEP-1 and Hep3B cells.
