# Supplementary material for: TTC36-Mediated Tumor Suppression via YBX3/SPRED1 Axis Paradoxically Reduces Sorafenib Sensitivity in Hepatocellular Carcinoma
Source: Int J Biol Sci. 2025 Oct 1;21(14):6351–72. doi: 10.7150/ijbs.115727 (PMC12594589; doi:10.7150/ijbs.115727)
Supplement: Supplementary file 2 — Supplementary tables. [file ijbsv21p6351s2.pdf]

Supplementary Table S1. Detailed clinical characteristics of the HCC patients

Features		Number of cases(n=80)	$TTC36^{low}$ (n=40)	$TTC36^{high}$ (n=40)	*P* value
Age(years)	<60	35	17	18	0.828
	≥60	45	23	22	
Gender	Male	52	24	28	0.248
	Female	28	16	12	
Tumor size (cm)	<5	66	28	38	**0.003**
	≥5	14	12	2	
T stage	I-II	55	20	35	**<0.001**
	III	25	20	5	
Histological differentiation	Well	60	22	38	**<0.001**
	Poor	20	18	2	
AFP (μg /L)	<20	16	10	6	0.197
	≥20	64	30	34	

Abbreviation: AFP, alpha fetoprotein

Supplementary Table S2. List of primers of qRT-PCR used in this study

Gene	Forward Primer	Reverse Primer
TTC36	ATAAAGCCGTCGGGCCTCAC	TCTGATGAGCACCGCAGTCC
SPRED1	GAGGGAGTGGACTAAGCAGC	CCTCTATCAAAAGCCCTAGCATC
YBX3	ACCGGCGTCCCTACAATTAC	GGTTCTCAGTTGGTGCTTCAC
GAPDH	AATCCCATCACCATCTTC	AGGCTGTTGTCATACTTC

Supplementary Table S3. List of antibodies used in this study

Antibody	Company
TTC36	Abcam
SPRED1	Abcam
phospho-MEK 1/2 ()	Abcam
MEK 1/2	Abcam

phospho-ERK 1/2	Abcam
ERK 1/2	Abcam
YBX3	Proteintech
His	Proteintech
Flag	Proteintech
HA	Proteintech
p38	Abcam
phospho-p38	Abcam
JNK	Proteintech
Phosphor-JNK	Proteintech
PI3K	Abcam
Phosphor-PI3K	Abcam
Akt	Abcam
Phosphor-Akt	Abcam
JAK	Abcam
Phosphor-JAK	Abcam
STAT3	Abcam
Phosphor-STAT3	Abcam
GAPDH	Proteintech

Supplementary Table S4. Sequence of sh-RNAs used in this study

shTTC36#1	GCCATCATCATCGACGTCAT-TTCAAGAGA-ATGACGTCGATGATGATGGC
shTTC36#2	CATCATCGACGTCATGATGA-TTCAAGAGA-TCATCATGACGTCGATGATG
shTTC36#3	CGACGTCATGATGATGATGA-TTCAAGAGA-TCATCATCATCATGACGTCG
shSPRED1#1	GCTGAAGCTGTACCTGAAT-TTCAAGAGA-ATTCAGGTACAGCTTCAGC
shSPRED1#2	GCCAAGATCTTCGAGATCAT-TTCAAGAGA-TGATCTCGAAGATCTTGGC
shSPRED1#3	GGACATCTACAGCTTCATC-TTCAAGAGA-GATGAAGCTGTAGATGTCC
shYBX3#1	GAGCGAGGAGCCCAAGAGAG-TTCAAGAGA-CTCTCTTGGGCTCCTCGCTC
shYBX3#2	GCCGCCTCCGCCGCGCGACC-TTCAAGAGA-GGTCGCGCGGCGGAGGCGGC
shYBX3#3	GAGGAGGAGGAGGCATGAGT-TTCAAGAGA-ACTCATGCCTCCTCCTCCTC
